# Gold Is Not Palladium: Linear Selectivity in Gold-Catalyzed Heck-Type Arylation via an Allylic Deprotonation–Coupling (ADC) Mechanism

**DOI:** 10.1021/acs.inorgchem.5c03734

**Published:** 2025-10-29

**Authors:** Kaveh Farshadfar, Robert Stranger, Alireza Ariafard, Kari Laasonen

**Affiliations:** † Department of Chemistry and Material Science, School of Chemical Engineering, 174277 Aalto University, Espoo 02150 Finland; ‡ Research School of Chemistry, 2219 Australian National University, Canberra 2601 Australian Capital Territory, Australia

## Abstract

Gold-catalyzed arylation of alkenes has recently been reported by Patil and co-workers as a rare example of a Heck-type reaction exhibiting exclusive linear selectivity. However, no mechanistic rationale was provided for the complete suppression of the branched product. In this study, we address this gap through DFT calculations and show that if the reaction proceeds via the classical Heck-type pathway, formation of the branched product is energetically preferred, which is inconsistent with experimental observations. This discrepancy led us to identify a distinct mechanistic alternative: an allylic deprotonation–coupling (ADC) mechanism, in which alkene–gold­(III) complexes undergo early allylic C–H deprotonation followed by C–C reductive elimination. The ADC mechanism is found to be energetically more favorable than the Heck pathway and accounts for the observed regioselectivity. This mechanism is also shown to be energetically far more favorable than an alternative involving nucleophilic attack by triflate, proposed for this catalytic transformation by Budzelaar et al. Comparative analysis further highlights the decisive role of the metal center in determining the reaction pathway: gold supports the ADC mechanism, while palladium promotes the conventional Heck-type process. The discovery of the ADC mechanism broadens the mechanistic understanding of transition-metal catalysis and provides a new perspective for future transformations.

## Graphical abstract

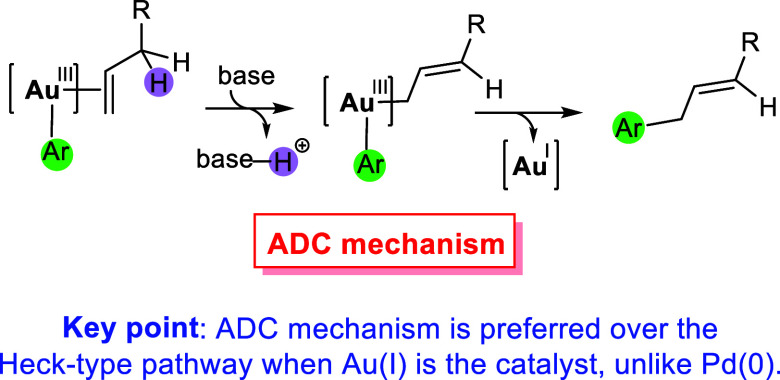

## Introduction

Heck-type reactions represent one of the most powerful transition metal-catalyzed methods for carbon–carbon bond formation, enabling efficient arylation of alkenes in a broad range of contexts.^1–3^ Although the original Heck reaction was developed using palladium catalysts, subsequent studies have shown that other transition metals such as Ni,^4–7^ Cu,^8–10^ and Ru,^11,12^ can also catalyze analogous alkene arylation reactions effectively.

These Heck-type reactions typically afford a mixture of linear (β) and branched (α) products, with the regioselectivity governed by the substrate and the catalyst environment.^13–16^ Figure 1a illustrates the canonical catalytic cycle for the Heck reaction, highlighting the elementary mechanistic steps that lead to the formation of both linear and branched arylated products. Accordingly, the reaction is initiated by oxidative addition of an aryl halide (Ar–X) to a Pd(0) species, affording the Pd­(II)–aryl complex **ii**. The alkene substrate then coordinates to the Pd­(II) center to form the π-complex **iii**, an intermediate which serves as the branching point for the two regioisomeric insertion pathways. If 2,1-insertion is energetically more favorable, the reaction proceeds to form the Pd­(II)–alkyl intermediate **iv**, from which a β-hydride elimination yields the linear (β) product **v**. Alternatively, if 1,2-insertion is preferred, the reaction proceeds through Pd­(II)–alkyl intermediate **vii**, from which β-hydride elimination furnishes the branched (α) product **viii**. Both pathways converge at the Pd­(II)–hydride complex **vi**, which undergoes reductive elimination to release HX and regenerate the active Pd(0) catalyst. The base serves to neutralize the HX formed in this final step, which renders the transformation exergonic and, in turn, facilitates catalyst turnover while preserving the overall efficiency of the reaction.

**Figure 1:**
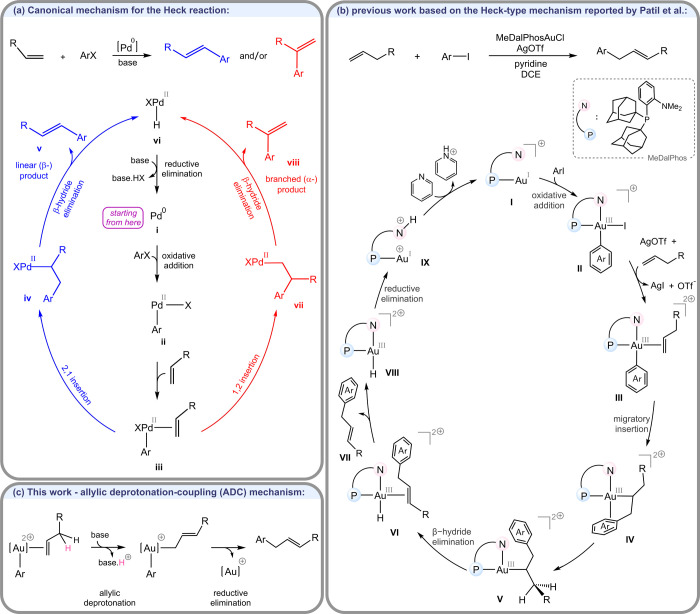
(a) Canonical catalytic cycle for the classical Heck reaction, highlighting the competing 2,1- and 1,2-insertion pathways that lead to the linear and branched products, respectively. (b) Proposed mechanism for the gold-catalyzed arylation of alkenes reported by Patil and co-workers. (c) Alternative mechanism introduced in this work, involving allylic deprotonation followed by C–C coupling via reductive elimination.

In alkene arylations via the Heck reaction, the linear product is generally more desirable from a synthetic and industrial perspective. As a result, considerable effort has been devoted to designing catalyst systems that favor selective formation of this regioisomer. In line with this goal, Patil and co-workers reported recently a rare example of exclusive linear selectivity in a gold-catalyzed Heck-type arylation of alkenes, enabled by a hemilabile (P,N) ligand in a Au­(I)/Au­(III) catalytic system (Figure 1b).^17,18^ Based on their interpretation that the reaction proceeds via a Heck-type mechanism, Patil and co-workers proposed the catalytic cycle shown in Figure 1b. This mechanism closely parallels the canonical pathway discussed above (Figure 1a) and begins with oxidative addition of the aryl iodide to the Au­(I) center, assisted by the hemilabile (P,N) ligand, followed by Ag­(I)-mediated iodide abstraction, alkene coordination, 2,1-migratory insertion, β-hydride elimination, and finally reductive elimination to regenerate the Au­(I) catalyst, with the resulting proton subsequently neutralized by the employed pyridine base.

Despite classifying the reaction as Heck-type, the authors did not explain why the branched product is completely suppressed and only the linear product is observed. In this study, one of our aims is to address this gap by using density functional theory (DFT) calculations to examine the mechanistic pathways that lead to the formation of both regioisomeric products.

Interestingly, this study shows that if the title reaction were to proceed through a Heck-type mechanism, the formation of the branched product would be significantly more favorable than that of the linear one. This outcome stands in clear contradiction to the experimental observation of exclusive linear selectivity and therefore suggests that the reaction must proceed through a mechanism distinct from the conventional Heck pathway.

In this study, we identify and examine this alternative mechanism in detail. Under this pathway, once the alkene–gold complex **III** (Figure 1c) is formed, it undergoes allylic C–H deprotonation, a process predicted to be significantly faster than either 1,2- or 2,1-migratory insertion. This deprotonation yields an allyl–gold­(III) complex, from which C–C coupling through a reductive elimination furnishes the final product. We designate this pathway as the Allylic Deprotonation–Coupling (ADC) mechanism.

At this juncture, it is worth noting that Budzelaar et al. also proposed an alternative mechanism for this Heck-type transformation catalyzed by gold, termed “triflate-induced nucleophilic attack,” in which OTf^–^ acts as the nucleophile attacking the alkene activated by the gold­(III) complex, thereby bypassing the migratory insertion step.^19^ Although Patil et al. questioned the validity of this pathway by replacing OTf^–^ with much weaker nucleophiles such as SbF_6_^–^ and BF_4_^–^, and still obtained the same product in comparable yields,^20^ we have nevertheless examined this mechanism computationally. Our results show that it is considerably less favorable than the ADC mechanism introduced in this study.

As a result, this work, by introducing the ADC mechanism, offers a novel perspective on gold-catalyzed reactivity and establishes a previously unrecognized route for C–C bond formation that challenges established mechanistic conventions.

## Results and Discussion

To investigate the mechanistic details of the title transformation, we employed density functional theory (DFT) calculations at the SMD/B3LYP-D3/def2-TZVP//SMD/B3LYP-D3/SDD, 6–31G­(d) level of theory in dichloroethane. To assess the robustness of our computed results, benchmark single-point energy calculations were performed on key intermediates and transition states using a range of functionals, including M06, M06-D3, M06L, M06L-D3, and ωB97XD, with the def2-TZVP basis set and SMD solvation model. The relative free energies of all key calculated transition structures based on these benchmark calculations are summarized in Table S1.

We initiated our investigation from the alkene–gold­(III) intermediate **III** (Figure 1b) to examine the feasibility of a Heck-type reaction and its potential regioselectivity toward either the linear or branched product. To this end, 1-hexene was selected as a representative alkene substrate, consistent with prior computational studies in this context.^17^ In a classical Heck-type mechanism, C–C bond formation proceeds via alkene insertion into the metal–aryl bond. Figure 2a illustrates the computed free energy profile comparing the two possible regioselective insertion pathways. In this profile, structure **1** (Figure 2b) is taken as the reference point, based on the observation that the external base, pyridine, binds significantly more strongly to Au­(III) than the substrate 1-hexene. Our DFT calculations reveal that the overall free energy barriers for 2,1-insertion (**TS**_**2–3**_) and 1,2-insertion (**TS**_**2–6**_) are 34.2 and 29.0 kcal/mol, respectively. The 2,1-insertion pathway leads to intermediate **3**, while 1,2-insertion affords intermediate **6**. In both intermediates, the aryl group engages in a stabilizing π-interaction with the Au­(III) center.

**Figure 2:**
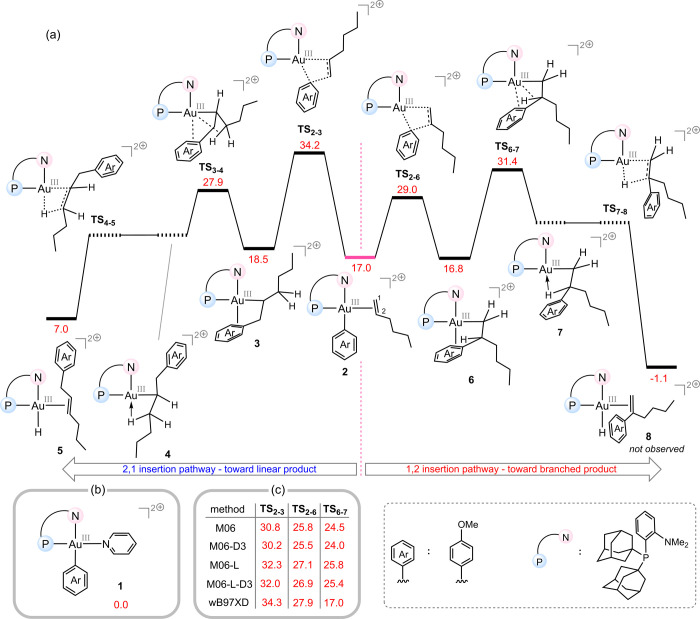
(a) Computed free energy profile for the classical Heck-type pathway starting from intermediate 2. The 1,2-insertion route is predicted to be more favorable than the 2,1-insertion route, in contrast with experimental observations. (b) Energy reference structure used in the calculations. (c) Benchmark calculations at different levels of theory confirming that the 1,2-insertion pathway consistently exhibits lower activation barriers than the 2,1-insertion pathway. The relative Gibbs free energy values are given in kcal/mol.

The next step in a typical Heck-type mechanism is β-hydride elimination. For this to occur, a ligand exchange must take place in which the coordinated aryl group is displaced by the β-C–H bond, allowing formation of agostic intermediates **4** and **7**. As shown in Figure 2a, these ligand exchange processes occur through transition structures **TS**_**3–4**_ and **TS**_**6–7**_. Intrinsic reaction coordinate (IRC) calculations for these two transition structures, followed by full optimization of the resulting geometries, were expected to yield agostic intermediates **4** and **7**. However, neither of these species corresponds to a true energy minimum; instead, both pathways proceed directly and spontaneously to the hydride complexes **5** and **8**. This behavior may be attributed to the strong affinity of the Au­(III) center for a hydride ligand, which stabilizes the Au–H bond and renders the agostic intermediates energetically inaccessible.

As shown in Figure 2a, both transition structures along the 1,2-insertion pathway (**TS**_**2–6**_ and **TS**_**6–7**_) lie energetically below **TS**_**2–3**_, which corresponds to the highest energy point along the competing 2,1-insertion pathway. This indicates that the 1,2-insertion pathway is energetically more favorable than the 2,1-insertion alternative. To ensure that this prediction is not dependent on the choice of functional, we computed the relative free energies of **TS**_**2–6**_, **TS**_**6–7**_, and **TS**_**2–3**_ based on single-point energies using several widely used DFT methods with the def2-TZVP basis set (Figure 2c). All functionals tested consistently reproduced the same trend, confirming that the 1,2-insertion pathway is significantly more favorable across different levels of theory. As a result, these computational results predict the formation of the branched product as the preferred outcome, rather than the linear one. However, this prediction is not consistent with the experimental findings of Patil et al., who reported that such catalytic reactions exclusively generate the linear product. In other words, if the reaction were to proceed through a classical Heck-type mechanism, our calculations clearly indicate that the branched product would be favored. The underlying reasons why the 1,2-insertion pathway is markedly more favorable than the 2,1-insertion pathway in the context of Au­(III) are discussed in detail in the Supporting Information. This discrepancy between computation and experiment suggests that an alternative, non–Heck-type mechanistic pathway must exist to account for the exclusive formation of the linear product. In the following section, we examine this alternative, termed the allylic deprotonation–coupling (ADC) mechanism, as introduced in the Introduction and illustrated in Figure 1c.

### Allylic Deprotonation–Coupling (ADC) Mechanism

The ADC mechanism is inspired by our previous work, in which we demonstrated that alkenes bearing allylic hydrogens become significantly more acidic upon coordination to electron-deficient transition metal centers.^21,22^ This enhanced acidity arises primarily from the stability of the allyl anion generated upon deprotonation, which increases with higher oxidation states and heavier metal centers. As a result, third-row transition metals, particularly in high oxidation states, stabilize the allyl anion more effectively, thereby enhancing the Bro̷nsted acidity of the coordinated alkene’s allylic C–H bond. In this regard, Au­(III) stands out as an exceptionally effective center: its combination of high oxidation state and third-row character enables strong interaction with the allyl anion, resulting in unusually acidic allylic hydrogens.

Figure 3a presents the computed free energy profile for the ADC mechanism, starting from π-complex **2**. Our calculations indicate that the triflate anion (OTf^–^) can serve as an effective base in this system, abstracting the allylic proton from intermediate **2** by overcoming an overall activation free energy of 22.5 kcal/mol, thereby generating species **9** and triflic acid (HOTf). Following the formation of HOTf, proton transfer to pyridine regenerates the triflate anion and further stabilizes the system by 15.3 kcal/mol. With this proton transfer complete, the system is poised to undergo the C–C coupling step. This process proceeds through the reductive elimination transition state **TS**_**9–10**_, which has a relative free energy of 16.9 kcal/mol, and is thermodynamically favorable, with an overall reaction free energy change of −26.4 kcal/mol. A subsequent coordination shift from the phenyl ring to the alkene double bond leads to a more stable species (**11**), with a calculated relative free energy of −30.9 kcal/mol. The overall exergonicity of −30.9 kcal/mol in Figure 3 reflects the net free energy change for the reaction **1** + alkene substrate → **11** + pyridine·H^+^, rather than product binding, and our calculations confirm that product release and continuation of the catalytic cycle remain energetically feasible (For details see the SI.)

**Figure 3:**
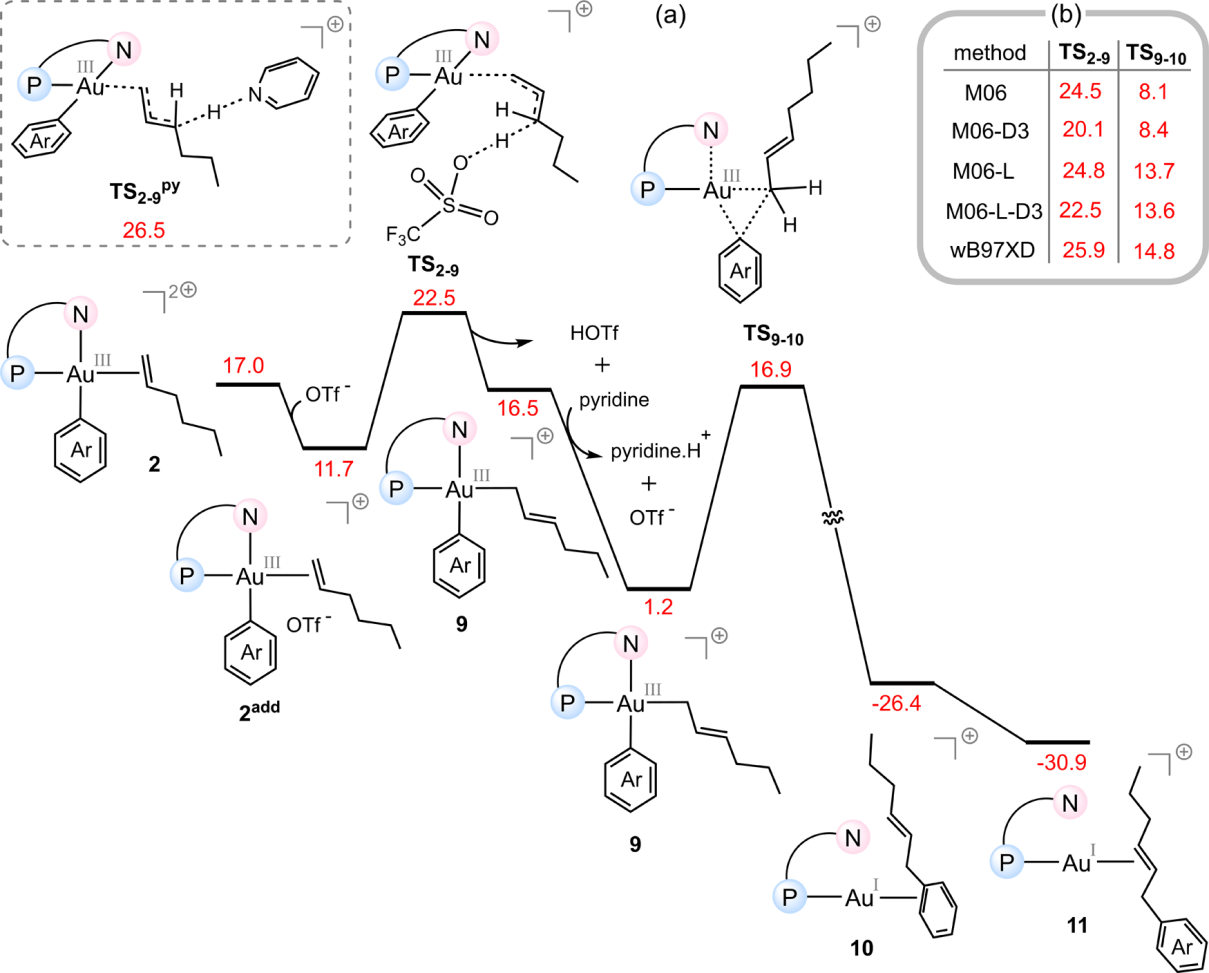
(a) Computed free energy profile for the ADC mechanism starting from π-complex **2**. (b) Benchmark calculations at various levels of theory for the relative free energies of key transition structures. The relative free energies are given in kcal/mol.

We also investigated the possibility that the ADC mechanism could generate the branched product and found that, in contrast to the Heck mechanism, the ADC pathway strongly favors formation of the linear product rather than the branched one (for details, see the Supporting Information).

At this juncture, it is important to note that although pyridine is a thermodynamically stronger Bro̷nsted base than the OTf^–^ anion, our calculations indicate that OTf^–^ acts as a more kinetically competent proton abstractor in the catalytic system under investigation. This behavior can be attributed to the dicationic nature of intermediate **2**, which creates a strong electrostatic attraction between the gold­(III) complex and the OTf^–^ anion, rendering their association energetically more favorable. As a result, OTf^–^ is better positioned for proton abstraction than pyridine, despite its weaker intrinsic basicity.

It is noteworthy that the highest energy point along the ADC mechanism corresponds to **TS**_**2–9**_ (22.5 kcal/mol), which lies well below both **TS**_**2–6**_ (29.0 kcal/mol) and **TS**_**6–7**_ (31.4 kcal/mol), the two highest-energy transition structures in the 1,2-insertion Heck-type pathway. Even in the absence of OTf^–^, allylic deprotonation of complex **2** by pyridine proceeds with an activation barrier of 26.5 kcal/mol, which also remains substantially lower than those associated with the insertion-based Heck pathways. This energetic comparison clearly demonstrates that the ADC mechanism, which leads to the experimentally observed product, is significantly more favorable than the Heck-type alternative. This conclusion is further supported by benchmark calculations performed at different levels of theory (Figure 3b). These additional calculations consistently show that **TS**_**2–9**_ lies above **TS**_**9–10**_, confirming **TS**_**2–9**_ as the rate-determining transition structure of the ADC mechanism, and that it remains lower in energy than both **TS**_**2–6**_ and **TS**_**6–7**_, further validating the energetic superiority of the ADC mechanism over the Heck-type pathway. Thus, since the allylic deprotonation in the proposed ADC mechanism is the rate-determining step, the calculated kinetic isotope effect (KIE) provides a testable prediction for the validity of this mechanism. According to our calculations, the KIE is 6.74 at 298 K, where both allylic hydrogens were replaced by deuterium

It is important to note that when BF_4_^–^ is used as the counterion instead of OTf^–^, it cannot act as a base, as our calculations show that deprotonation of the allylic hydrogen then proceeds with a high free energy barrier of 36.1 kcal/mol. For completeness, we have also computationally examined whether other anions such as ClO_4_^–^, HCO_3_^–^, NO_3_^–^, OTs^–^, and TFA^–^ can serve as replacements for OTf^–^ and found that OTf^–^ remains the most suitable choice. Details of this analysis are provided in the Supporting Information.

### Metal-Dependent Divergence between ADC and Heck Pathways

As discussed above, starting from an Au­(I) catalyst and employing substrates typical of Heck-type arylations, the transformation preferentially proceeds through the ADC mechanism rather than the classical Heck pathway. This raises an important mechanistic question: what happens if the Au­(III) center in key intermediate **2** (Figure 3a) is replaced with Pd­(II) to generate the analogous intermediate **13** (Figure 4a)? Would the system still favor the ADC pathway, or would it instead proceed preferentially through the Heck-type mechanism?

**Figure 4:**
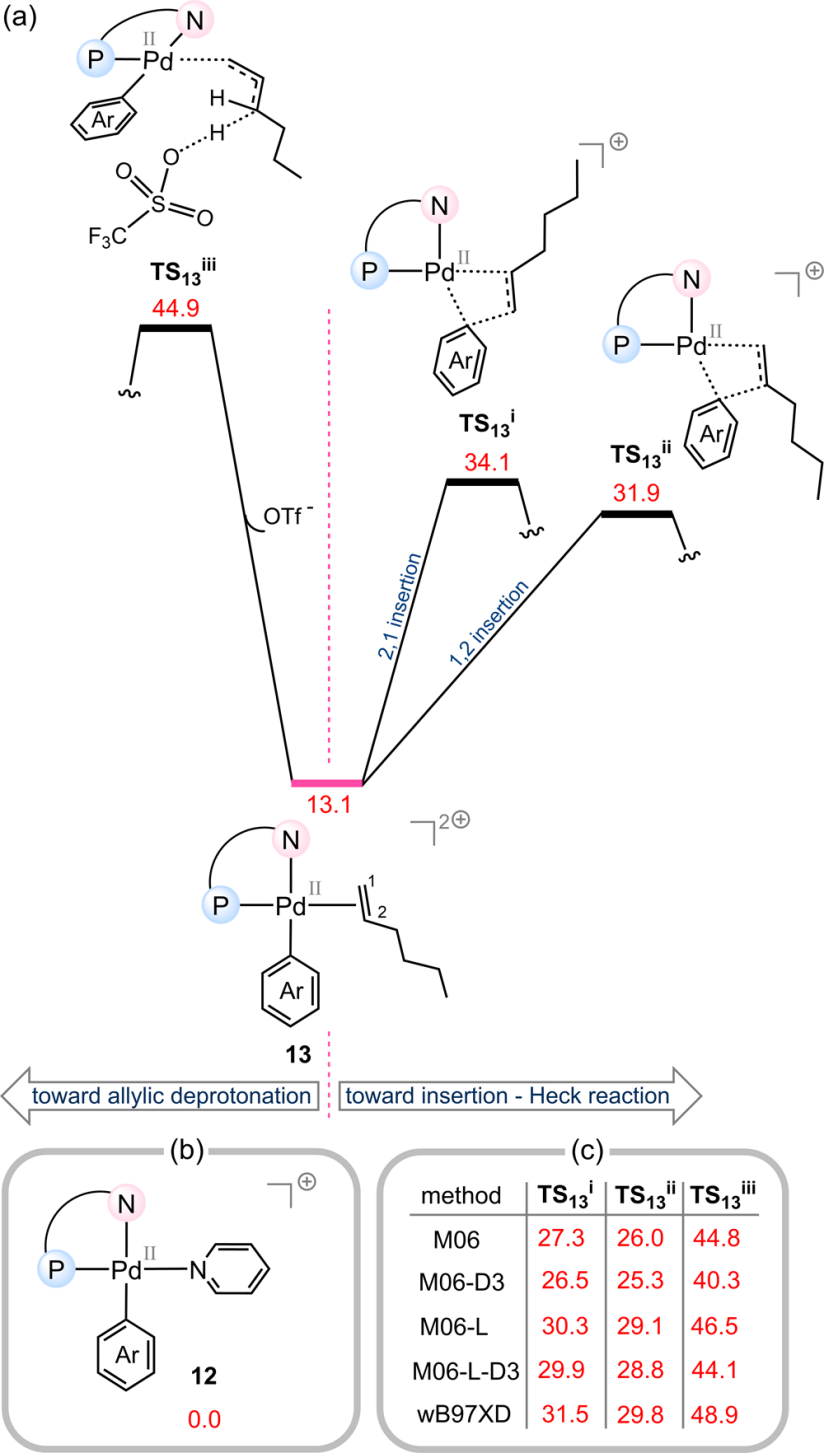
(a) Comparison of energy barriers for Heck-type and ADC pathways when Au­(III) is replaced with Pd­(II), starting from the Pd π-complex **13**. (b) Energy reference structure used in these calculations. (c) Benchmark DFT calculations confirming that the ADC pathway is disfavored for the Pd­(II) system, consistent with a return to classical Heck-type reactivity. The relative free energies are given in kcal/mol.

Figure 4a compares the relative free energies of the key transition states associated with both the Heck and ADC mechanisms, using the Pd­(II)–pyridine complex **12** (Figure 4b) as the reference point. This straightforward comparison clearly demonstrates that under these modified conditions, the Heck pathway becomes considerably more favorable than the ADC alternative, as evidenced by the fact that both the 2,1- and 1,2-insertion transition states (**TS**_**13**_^**i**^ and **TS**_**13**_^**ii**^) lie significantly lower in energy than the corresponding allylic deprotonation transition state (**TS**_**13**_^**iii**^). This conclusion is further supported by relative free energies obtained from additional single-point calculations using alternative functionals beyond B3LYP-D3, as shown in Figure 4c.

This finding is consistent with precedent in the literature, which has long established that palladium catalysis typically proceeds through the classical Heck mechanism, as discussed in the Introduction (Figure 1a). Moreover, it demonstrates that the nature of the metal center, gold versus palladium, plays a decisive role in determining the operative mechanism, whether it follows the ADC pathway or the classical Heck pathway.

A comparison of the three free energy profiles (Figures [Fig fig2]2a, 3a, and 4a) indicates that changing the metal center from Au­(III) to Pd­(II) has minimal impact on the overall activation barriers for the 1,2- and 2,1-insertion pathways associated with the Heck mechanism. However, this is not the case for the allylic deprotonation step in the ADC mechanism: the activation barrier for deprotonation with Au­(III) is 22.5 kcal/mol, whereas with Pd­(II) it rises sharply to 44.9 kcal/mol. This highlights how strongly the acidity of the allylic hydrogen of the coordinated alkene substrate is influenced by the nature of the metal center. Au­(III), a third-row transition metal in a + 3 oxidation state, renders the allylic hydrogen significantly more acidic than Pd­(II), a second-row transition metal in a + 2 oxidation state. This difference in electronic character makes deprotonation in the gold system far more favorable than in the palladium system, thereby enabling the catalytic reaction to proceed through the ADC mechanism.

In agreement with the above results, our DFT calculations for complexes **2** and **13** show a pronounced difference in allylic hydrogen acidity: complex **2** exhibits a calculated aqueous p*K*_a_ of – 1.1, whereas complex **13** has a p*K*_a_ of 18.8, highlighting the substantially higher acidity of the allylic hydrogens in the Au­(III) complex compared to the Pd­(II) analogue.

### Computational Evaluation of the Budzelaar Mechanism

Following the publication of two studies by Patil and co-workers proposing gold-catalyzed Heck-type reactions,^17,18^ their mechanistic interpretation was subsequently challenged by Budzelaar et al.^19^ They argued that the transformation does not constitute a genuine gold-catalyzed Heck reaction, and instead proposed an alternative mechanism (Figure 5a), in which OTf^–^ acts as a nucleophile, attacking the C^2^ position of the coordinated alkene to form intermediate **X**. This is followed by alkyl–aryl reductive elimination to generate intermediate **XI**. A final deprotonation at C^3^ by OTf^–^, resulting in elimination of HOTf, was suggested to furnish the observed product **VII**. However, as discussed in the Introduction, this OTf^–^-induced nucleophilic attack mechanism was ruled out by Patil et al.,^20^ who clearly demonstrated that replacing OTf^–^ with much weaker nucleophiles such as SbF_6_^–^ and BF_4_^–^ still gave the same product in comparable yields.

**Figure 5:**
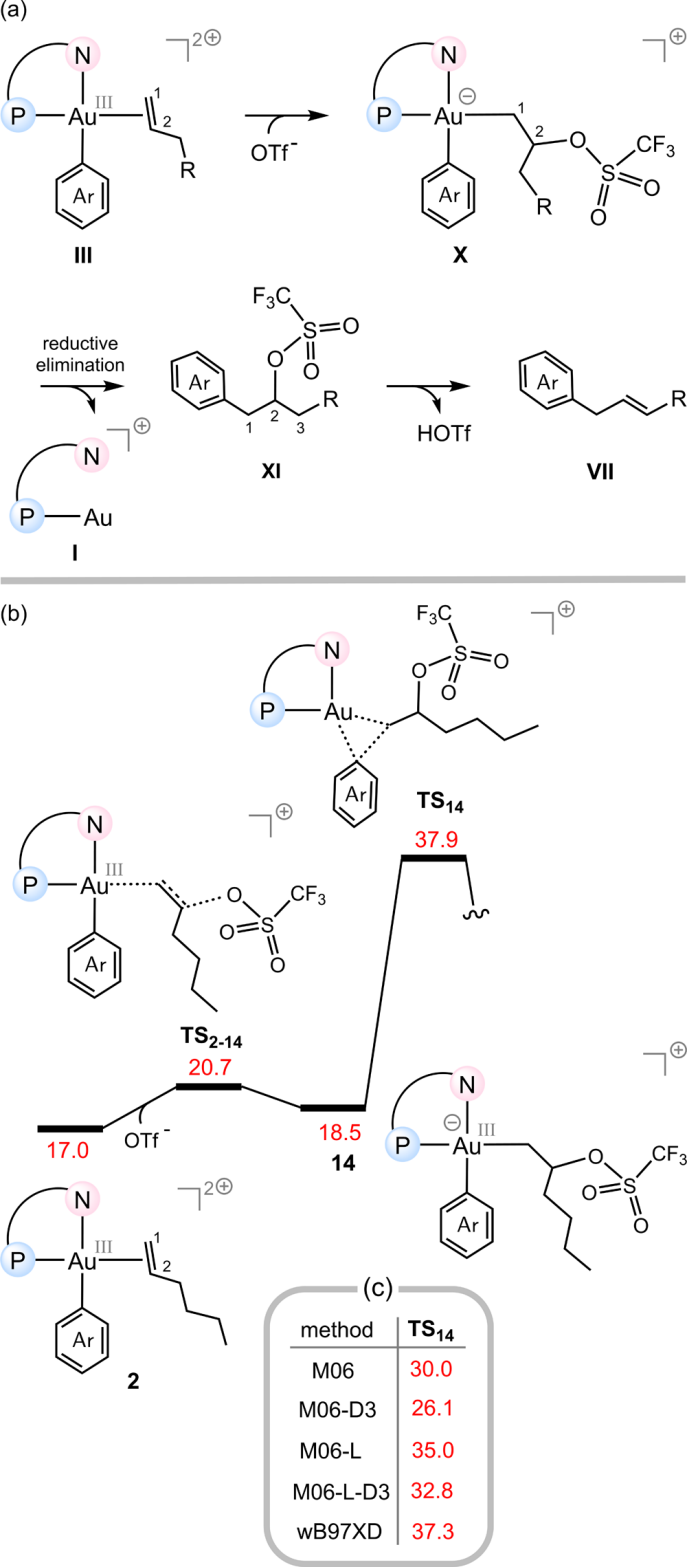
(a) Mechanistic proposal by Budzelaar and co-workers, featuring OTf^–^ attack at C^2^ followed by reductive elimination and deprotonation to yield the final product. (b) DFT-computed energy profile for the Budzelaar mechanism, showing a high energy barrier for the reductive elimination step. (c) Benchmark results validating that this mechanism is energetically less favorable than the ADC pathway. The relative free energies are given in kcal/mol.

We nevertheless investigated the mechanism proposed by Budzelaar and co-workers using DFT calculations for completeness (Figure 5b). While this pathway is theoretically capable of affording the observed product, our results show that the energy barrier for the reductive elimination step via transition structure **TS**_**14**_ is substantially higher than the highest barrier on the ADC mechanism (**TS**_**2–9**_, Figure 3a), making Budzelaar’s pathway unlikely to be operative. Further confirmation of this conclusion comes from benchmark computations (Figure 5c) performed across multiple levels of theory, all of which indicate that the mechanism proposed by Budzelaar et al. is energetically less favorable than the ADC mechanism described in this work.

### Proposed Catalytic Cycle for Gold-Catalyzed Arylation of Alkenes via the Allylic Deprotonation–Coupling (ADC) Mechanism

Taken together, our computational and mechanistic analysis supports a distinct catalytic cycle for the gold-catalyzed arylation of alkenes, which we term the allylic deprotonation–coupling (ADC) mechanism (Figure 6). The reaction begins with oxidative addition of Ar–I to the Au­(I) complex, facilitated by the hemilabile ligand. This is followed by ligand exchange, assisted by Ag^+^, to generate the key Au­(III)–alkene complex **III**. Subsequently, a base such as OTf^–^ or pyridine promotes allylic C–H deprotonation of the coordinated alkene, generating a stabilized allyl–gold­(III) intermediate that undergoes direct C–C reductive elimination. The overall process proceeds through low-energy barriers and accounts for the observed linear selectivity, while also rationalizing the ineffectiveness of alternative pathways such as migratory insertion or triflate-induced nucleophilic attack.

**Figure 6:**
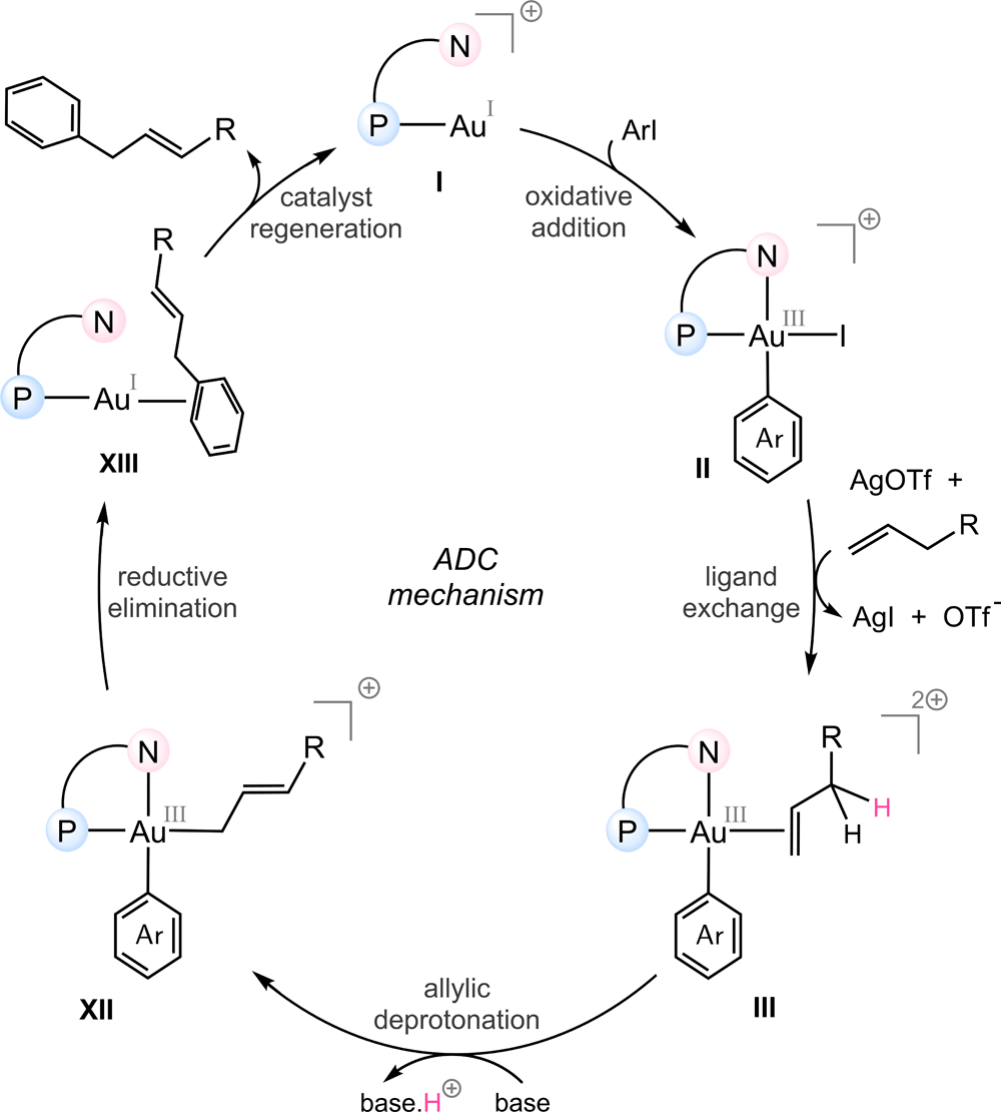
DFT-derived catalytic cycle proposed for gold-catalyzed alkene arylation through the ADC mechanism.

## Conclusions

This study demonstrates that the nature of the transition metal center can fundamentally alter the operative reaction mechanism, even when the overall transformation and product remain the same. While the gold-catalyzed reaction proceeds through a distinct allylic deprotonation–coupling (ADC) mechanism, the analogous palladium system follows the classical Heck pathway. This divergence arises from the markedly higher acidity of allylic hydrogens of the Au­(III)–coordinated alkene substrates, which facilitates deprotonation by a suitable base early in the catalytic cycle. These findings not only clarify the origin of linear selectivity in gold-catalyzed arylations but also underscore the broader principle that subtle changes in catalyst identity can redirect the entire mechanistic landscape of a reaction.

## Computational Details

Gaussian 16 ^23^ was used to fully optimize all the structures reported in this paper at the B3LYP level of theory.^24^ For all the calculations, solvent effects were considered using the SMD solvation model^25^ with dichloroethane as the solvent. The SDD basis set^26,27^ with effective core potential (ECP) was chosen to describe gold, while the 6–31G­(d) basis set was employed for all other atoms.^28^ This basis set combination will be referred to as BS1. We also employed Grimme’s empirical dispersion (D3) correction for all the calculations. Frequency calculations were carried out at the same level of theory as those for the structural optimization. Transition structures were located using the Berny algorithm. IRC calculations were used to confirm the connectivity between transition structures and minima.^29,30^ To further refine the energies obtained from the SMD/B3LYP-D3/SDD,6-31G­(d) calculations, we carried out single-point energy calculations using the B3LYP-D3 functional method with the SMD solvation model in dichloroethane along with a larger basis set (BS2) for all the optimized structures. BS2 corresponds to the def2-TZVP basis set^31^ on all atoms, with the associated def2-ECP applied to gold. The tight convergence criterion and ultrafine integral grid were exploited to increase the accuracy of the calculations. The free energy for each species in solution was calculated using the following formula:

$$G=E(\mathrm{BS}2)+G(\mathrm{BS}1)-E(\mathrm{BS}1)+\Delta G^{1\mathrm{atm}\to 1M}$$ 1

where Δ*G* ^1atm→1M^ = 1.89 kcal/mol is the free-energy change for compression of 1 mol of an ideal gas from 1 atm to the 1 M solution phase standard state.

## Supplementary Material



## Notes

Additional computational results; Cartesian coordinates of all calculated species (PDF)
