# Supplementary material for: Gold Is Not Palladium: Linear Selectivity in Gold-Catalyzed Heck-Type Arylation via an Allylic Deprotonation–Coupling (ADC) Mechanism
Source: Inorg Chem. 2025 Oct 29;64(44):22030–8. doi: 10.1021/acs.inorgchem.5c03734 (PMC12606697; doi:10.1021/acs.inorgchem.5c03734)
Supplement: Supplementary file 1 [file ic5c03734_si_001.pdf]

Supporting Information for:

Gold Is Not Palladium: Linear Selectivity in Gold-Catalyzed Heck-Type Arylation via an Allylic Deprotonation-Coupling (ADC) Mechanism

Kaveh Farshadfar[1,*], Robert Stranger[2], Alireza Ariafard[2,*] and Kari Laasonen[1,*]

[1]Department of chemistry and material science, School of chemical engineering, Aalto University, 02150 Espoo, Finland

[2]Research School of Chemistry, Australian National University, Canberra, Australian Capital Territory 2601, Australia

Corresponding Authors

Kaveh Farshadfar — Email: Kaveh.Farshadfar@Aalto.fi

Kari Laasonen — Email: Kari.Laasonen@Aalto.fi

Alireza Ariafard — Email: Alireza.Ariafard@anu.edu.au

Content:

Origin of 1,2-insertion preference in gold catalysis (page S2)

Investigation of possible branched pathway in the ADC mechanism (page S2)

Catalyst turnover and product release (page S3)

Table S1. Relative free energies (kcal/mol) of key transition states obtained with different DFT functionals. (page S5)

Effect of anion identity and basicity on the allylic deprotonation step (page S5)

Table S3. Cartesian coordinates and total energies for all of the calculated structures. (page S8)

Origin of 1,2-Insertion Preference in Gold Catalysis

The marked preference for the 1,2-insertion pathway over the 2,1-insertion in the gold complex can be rationalized by considering the high electron deficiency of the Au(III) center. This feature enables significant π-donation from the alkene to the gold center, thereby increasing the contribution of resonance structure **b** (Figure S1), in which electron density from the alkene is delocalized toward gold.

This interpretation is supported by the substantial partial positive charge (+0.271) calculated on the 1-hexene fragment, as well as the shorter Au–C^1 bond relative to Au–C^2, indicating polarization of the π-system. The increased weight of resonance contributor **b** facilitates nucleophilic aryl attack at C^2, rendering the 1,2-insertion pathway energetically more favorable.

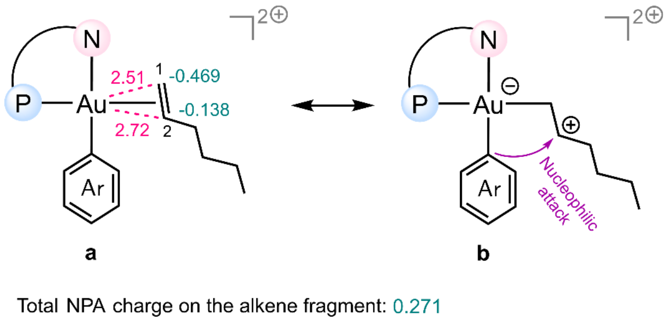

Figure S1. Gold-coordinated 1-hexene (a) and resonance structure (b) leading to 1,2-insertion. Selected bond distances are shown in pink (in Å), and natural population analysis (NPA) charges are shown in blue.

Investigation of Possible Branched Pathway in the ADC Mechanism

We investigated the possibility that the ADC mechanism could also generate the branched product. For this to occur, the η^1-allyl ligand in intermediate **9** must first rearrange to an η^3-allyl form (complex **16**, Figure S2). Conversion of the η^1-allyl species **9** to the η^3-coordinated form **16** requires decoordination of the nitrogen donor from the gold centre. Our DFT calculations show that this η^3-allyl intermediate lies 15.1 kcal/mol above **9**. This species can further rearrange to a new η^1-allyl complex **17** with a relative free energy of 6.1 kcal/mol. C–C reductive elimination from either complex **16** or **17** could, in principle, afford the branched product; however, these processes proceed via transition structures $\mathbf{TS_{16}}$ and $\mathbf{TS_{17}}$ with relative free energies of 23.5 and 23.9 kcal/mol, respectively.

Our calculations show that both **TS$_{16}$** and **TS$_{17}$** are significantly higher in energy than **TS$_{9\text{–}10}$** (16.9 kcal/mol), which leads to the linear product. These results demonstrate that the ADC pathway exclusively favours formation of the linear product, consistent with experimental observations, whereas the classical Heck mechanism is predicted to favour the branched product.

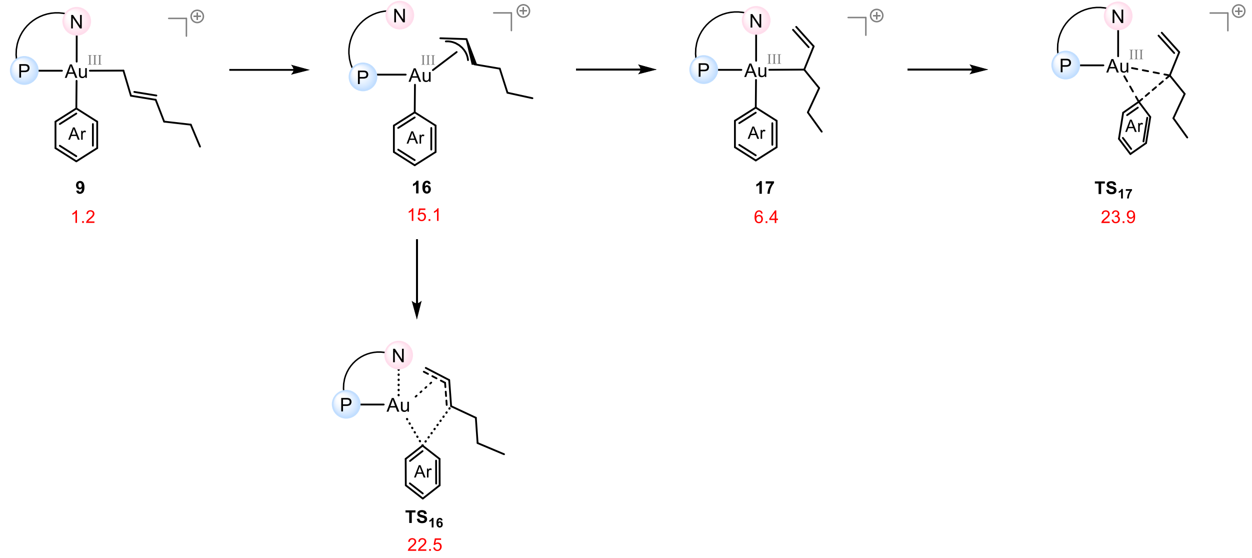

Figure S2. Calculated energy profile for the formation of the branched product via the Allylic Deprotonation–Coupling (ADC) mechanism. Relative Gibbs free energy values are given in kcal/mol.

Catalyst Turnover and Product Release

The negative free energy of ΔG = -30.9 kcal/mol reported in Figure 3 reflects the overall thermodynamic driving force associated with C–C bond formation, reduction of Au(III) to Au(I), proton transfer to pyridine, and other elementary steps (Figure S3), rather than an irreversible trapping of the catalyst by the product.

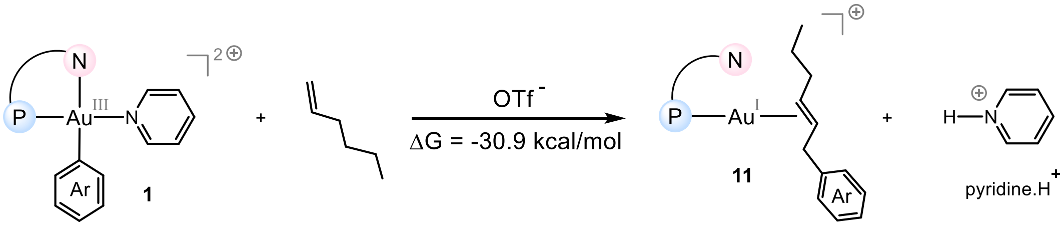

Figure S3. Overall free energy change (ΔG = –30.9 kcal/mol) for the OTf$^-$-mediated **1** + alkene substrate → **11** + pyridine·H$^+$.

To confirm that the catalyst remains active, we further examined (Figure S4) the first elementary step of the next catalytic cycle, namely oxidative addition of Ar–I (see Figure 6). For turnover to occur, the product must first dissociate and be displaced by Ar–I. Our calculations show that this ligand exchange is only slightly endergonic (ΔG = +5.6 kcal/mol), and the subsequent oxidative addition proceeds via **TS$_{15}$** with a moderate energy barrier of 19.5 kcal/mol. The resulting Au(III) complex **II** is slightly exergonic (ΔG = –0.6 kcal/mol). These results demonstrate that product release and catalyst re-entry into the next catalytic cycle are energetically accessible, and therefore the overall exergonicity of –30.9 kcal/mol depicted in Figure 3 should not be interpreted as catalyst inhibition.

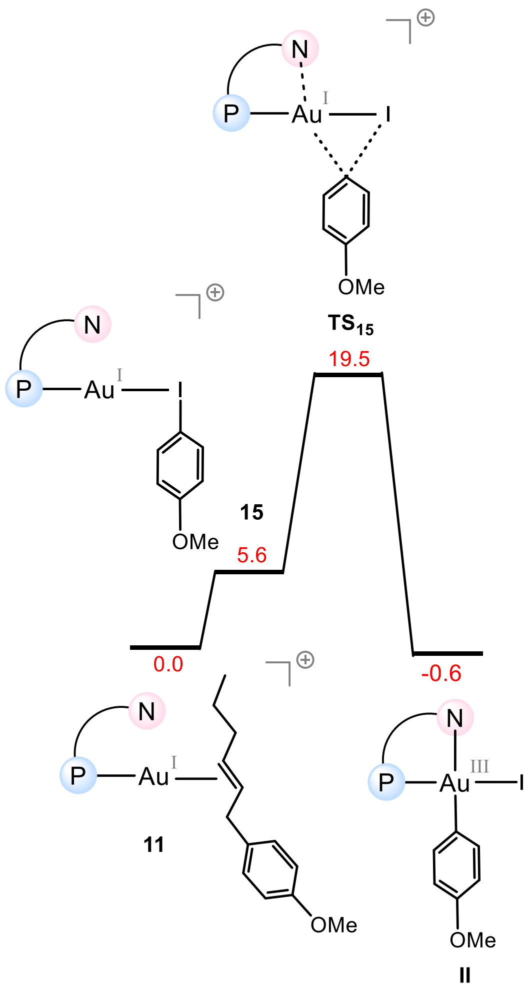

Figure S4. Calculated Gibbs free energy profile for regeneration of the active gold(III) complex **II** from gold(I) species **11**. Relative Gibbs free energy values are given in kcal/mol.

Table S1. Relative free energies (kcal/mol) of key transition states obtained with different DFT functionals.

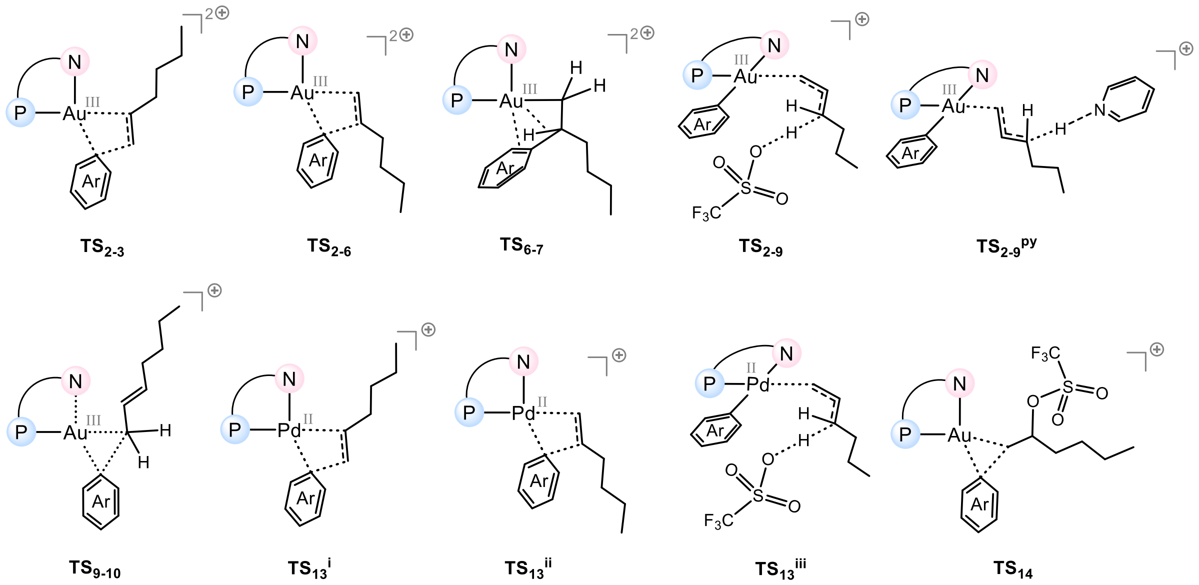

method	B3LYP-D3	M06	M06-D3	M06-L	M06-L-D3	ωB97XD
**$TS_{2\text{-}3}$**	34.2	30.8	30.2	32.3	32.0	34.3
**$TS_{2\text{-}6}$**	29.0	25.8	25.1	27.1	26.9	27.9
**$TS_{6\text{-}7}$**	31.4	24.5	24.0	25.8	25.4	17.0
**$TS_{2\text{-}9}$**	22.5	24.5	20.1	24.8	22.5	25.9
**$TS_{2\text{-}9}^{py}$**	26.5	26.9	23.8	26.3	24.5	27.0
**$TS_{9\text{-}10}$**	16.9	8.1	8.4	13.7	13.6	14.8
**TS_{13}**	34.1	27.3	26.5	30.3	29.9	31.5
**TS_{13}^{ii}**	31.9	26.0	25.3	29.1	28.8	29.8
**TS_{13}^{iii}**	44.9	44.8	40.3	46.5	44.1	48.9
**TS_{14}**	37.9	30.0	26.1	35.0	32.8	37.3

Effect of Anion Identity and Basicity on the Allylic Deprotonation Step

Patil and co-workers have already demonstrated experimentally that replacing OTf^- with BF_4^- as the counterion still affords catalytic turnover with comparable yields. In such cases, pyridine must operate as the actual base to promote allylic deprotonation. Consistent with this observation, our calculations show that OTf^- mediates the deprotonation with a barrier of 22.5 kcal/mol, while pyridine promotes the same step with a barrier of 26.5 kcal/mol (Figure 3). Furthermore, when BF_4^- itself is forced to act as the

deprotonating agent, the overall barrier rises to 36.1 kcal/mol, confirming that it is not kinetically competent.

To further strengthen this point and to establish a guideline for which anions can act as deprotonating agents in place of OTf^-, we extended our calculations to a series of representative oxyanions (A^-), namely ClO_4^-, HCO_3^-, NO_3^-, OTs^-, and TFA^-. For each case, we optimised the adduct between the anion A^- and the allylic hydrogen (structure **ii**), then located the corresponding transition structure for proton abstraction (**TS**$_{ii}$). The computed free energy difference between adduct **ii** and **TS**$_{ii}$ (ΔG^{act}) was found to correlate directly with the basicity of the oxyanion: the more basic the anion A^-, the lower the barrier for proton abstraction. Using the pK_a of the conjugate acid HA as a measure of basicity (smaller pK_a = weaker base), we obtained a clear linear correlation between ΔG^{act} and pK_a with $R^2 = 0.97$, confirming that more basic anions facilitate the deprotonation step more effectively (Figure S5).

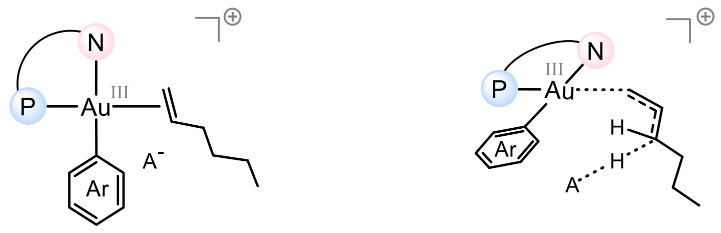

ii **TS**$_{ii}$

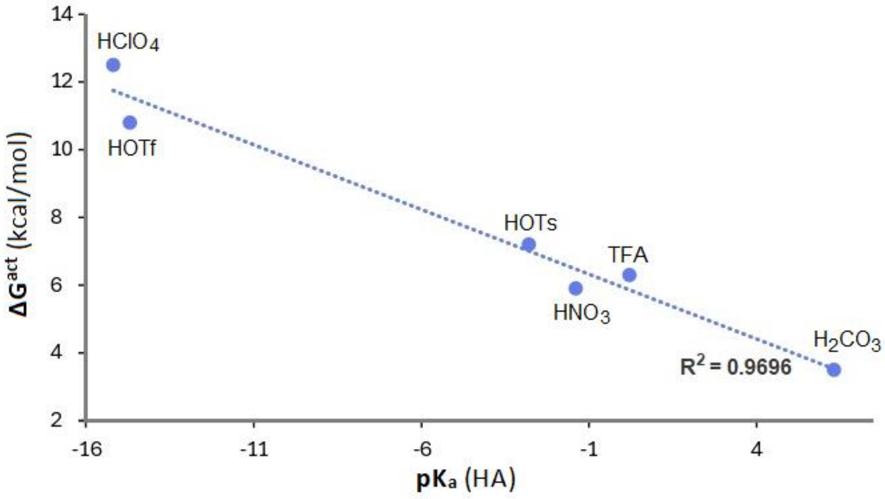

Figure S5. Relationship between the calculated activation free energy for proton abstraction (ΔG^{act}) and the conjugate acid pK_a of various oxyanions (A^-).

However, our analysis also shows that the story does not end here. More basic oxyanions, while promoting proton abstraction more easily, also tend to bind more strongly to the Au(III) centre. This stronger binding can raise the overall activation barrier for deprotonation. For example, although HCO_3^- is relatively basic and promotes proton abstraction efficiently, it binds much more strongly to Au(III) than pyridine, leading to an overall barrier of 30.3 kcal/mol. These results demonstrate that basicity alone does not determine catalytic competence: the ideal anion must not only be able to abstract the proton efficiently but also avoid excessively strong binding to Au(III). Within this balance, OTf^- emerges as the optimal choice, giving the lowest overall $\Delta G^‡$ for deprotonation and thus serving as the most effective proton abstractor in the catalytic system.

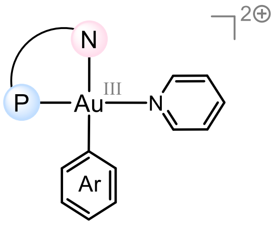

1

0.0

energy reference

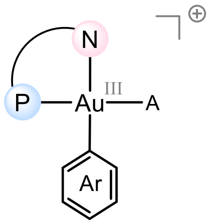

i

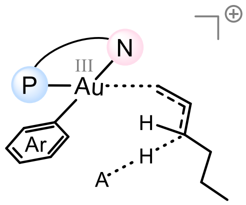

TS_{ii}

Table S2. Calculated activation energies for allylic deprotonation with various anions along with the corresponding pK_a values of HA.

A^-	**i**	**TS_{ii}**	$\Delta G^‡$	pK_a (HA)
OTf^-	0.4	22.5	22.5	-14.7
ClO_4^-	1.2	24.0	24.0	-15.2
OTs^-	-12.5	13.4	25.9	-2.8
NO_3^-	-9.0	16.4	25.4	-1.4
TFA^-	-11.9	17.0	28.9	0.2
HCO_3^-	-18.6	11.7	30.3	6.3

Table S3. Total potential (E), and Gibbs free energies (G) of all structures optimized at the SMD/B3LYP-D3/BS1 level of theory along with the total potential energies calculated by SMD/B3LYP-D3/BS2//SMD/ B3LYP-D3/BS1 and Cartesian coordinates for all of the calculated structures.

1

E (SMD/B3LYP-D3/BS1) = -2217.32610389 au
G (SMD/B3LYP-D3/BS1) = -2216.536277 au
E (SMD/B3LYP-D3/BS2//SMD/B3LYP-D3/BS1) = -2218.00435015 au

C	1.85505100	-1.58631100	-2.06216100
C	2.89261600	-1.20484200	-2.93693600
H	3.02814900	-0.16634900	-3.20314400
C	3.75921600	-2.14772100	-3.48115000
H	4.55110800	-1.82566700	-4.15001800
C	3.60115000	-3.49655900	-3.16466200
H	4.26936400	-4.24324100	-3.58268200
C	2.57639700	-3.89298800	-2.30977200
H	2.45870200	-4.94538500	-2.07844600
C	1.70461500	-2.94776600	-1.75723800
C	1.20190000	-4.16843100	0.29337000
H	1.75158500	-5.05063000	-0.04590300
H	0.38880900	-4.49401100	0.94183300
H	1.87309500	-3.50615400	0.84312200
C	-0.31307700	-4.29885800	-1.63352000
H	-0.74328200	-3.72646200	-2.45735000
H	-1.10512000	-4.63800900	-0.96515200
H	0.21290200	-5.17316200	-2.02840000
Au	-0.63325700	-1.73462000	-0.02264800
N	0.62554400	-3.41881000	-0.86666200
P	0.72537900	-0.32931700	-1.33795100
C	-1.86896700	-0.30243900	0.78152400
C	-3.03803900	0.07207900	0.12607000
C	-1.61918600	0.15192200	2.08206000
C	-3.95131500	0.93693900	0.74517000
C	-2.52183400	1.01109300	2.70212700
C	-3.69106600	1.41639100	2.03587100
H	-4.84973900	1.22189600	0.21005800
H	-2.33696400	1.37981500	3.70659000
H	-0.72416700	-0.14572300	2.61971600
H	-3.26387400	-0.28929000	-0.87156500
O	-4.49835600	2.26235900	2.72580000
C	-5.70109900	2.71389700	2.10101700
H	-5.48828100	3.27120900	1.17988300
H	-6.37385600	1.87675000	1.87563800
H	-6.17931300	3.37789900	2.82362500
C	-0.26197900	0.42681700	-2.77266300
C	-1.09063000	2.59322700	-4.63632100

H	-1.71415700	2.97275600	-5.45620300
H	-0.41997000	3.40763300	-4.33130900
C	-2.11989400	-0.16103800	-4.39757300
H	-2.79651600	-0.96612600	-4.70982600
C	-1.06188100	1.66855100	-2.30604600
H	-1.65474900	1.44232100	-1.41741600
H	-0.36681600	2.47162800	-2.04652500
C	-0.27248600	1.38171900	-5.11858700
H	0.38067100	1.67738900	-5.94855100
C	-1.23331500	-0.68951600	-3.25100100
H	-0.65808300	-1.56083100	-3.58984700
H	-1.87124700	-1.01616300	-2.42244900
C	-1.97344500	2.16482800	-3.44958700
H	-2.54708700	3.02337400	-3.07982600
C	-1.23012000	0.26634000	-5.57964000
H	-0.65880100	-0.59485400	-5.95142500
H	-1.85399900	0.62701500	-6.40751500
C	0.62536500	0.84648400	-3.97582700
H	1.34425800	1.61553800	-3.67165200
H	1.18484300	-0.01188600	-4.35623000
C	-2.93455900	1.04432400	-3.88908800
H	-3.59788000	1.40987600	-4.68341300
H	-3.57037500	0.74121800	-3.04602400
C	1.77238500	0.79789000	-0.22854300
C	2.31283300	1.80451000	2.51109600
H	2.90100200	2.38504000	3.23336400
H	1.48143800	1.34337500	3.06213500
C	3.80118400	2.29178600	0.00937700
H	4.62736900	2.74591000	-0.55098100
C	0.91255400	1.91777300	0.41130900
H	0.53960100	2.59371500	-0.36027100
H	0.04802800	1.49438400	0.92345900
C	3.19272900	0.71373400	1.87114900
H	3.58202700	0.04208600	2.64616300
C	2.95786400	1.45431200	-0.98228600
H	3.60670400	0.69003100	-1.41575300
H	2.58853000	2.09069400	-1.79444100
C	1.76694800	2.73068200	1.40873300
H	1.12604200	3.50142600	1.85387900
C	4.36079100	1.36615000	1.10694300
H	4.99837400	0.59162300	0.65966900
H	4.98536600	1.94314600	1.80093500
C	2.33891200	-0.11977400	0.89182000
H	1.51414200	-0.59454800	1.43950400
H	2.94864100	-0.91884900	0.45231000
C	2.93447200	3.38949500	0.65145500
H	3.54208100	3.99119300	1.33982500

H	2.54754300	4.06755300	-0.12104500
C	-2.93420200	-3.64105600	0.69540900
C	-3.74506300	-4.45175500	1.48042200
C	-3.39307800	-4.67276600	2.81270100
C	-2.23806600	-4.07342300	3.31763300
C	-1.47328400	-3.27390800	2.47784700
N	-1.81609100	-3.07349800	1.18917700
H	-4.01115500	-5.29860700	3.44865500
H	-3.16918200	-3.42988000	-0.34163000
H	-4.63607700	-4.89327400	1.04800800
H	-1.92927500	-4.21414800	4.34759300
H	-0.57261300	-2.78037800	2.82381100

2

E (SMD/B3LYP-D3/BS1) = -2204.86601354

G (SMD/B3LYP-D3/BS1) = -2204.004874

E (SMD/B3LYP-D3/BS2//SMD/B3LYP-D3/BS1) = -2205.54130746

C	2.40326300	-1.53630300	-0.95186400
C	3.78719100	-1.44930100	-1.21004400
H	4.34449800	-0.56959600	-0.92142600
C	4.46644600	-2.48386200	-1.84554200
H	5.53225800	-2.39207600	-2.02940900
C	3.77086000	-3.62307400	-2.24855300
H	4.28547700	-4.43262100	-2.75683500
C	2.40566500	-3.72792800	-1.99734400
H	1.87820300	-4.61835500	-2.32183500
C	1.72016100	-2.69871800	-1.34094200
C	0.04580600	-4.10711900	-0.28372300
H	0.45839000	-4.98249600	-0.79054900
H	-1.02590300	-4.25853100	-0.16535300
H	0.51923600	-3.99410200	0.69295800
C	-0.46834700	-2.94432000	-2.38190100
H	-0.24738500	-2.05504500	-2.97399300
H	-1.53881300	-2.99156000	-2.17491400
H	-0.17446400	-3.83846500	-2.94040900
C	1.55207900	1.27837100	-1.52099800
C	0.78075200	0.71956100	-2.74877300
H	-0.24841700	0.47104800	-2.46188900
H	1.25824300	-0.20012300	-3.10713600
C	0.75894700	1.77225200	-3.87723900
H	0.21317700	1.35032300	-4.73004600
C	0.86895600	2.57769100	-1.02441700
H	-0.14042000	2.37423000	-0.66662300
H	1.43188300	3.00614400	-0.19394800
C	2.96349800	2.68511600	-3.08187100
H	3.99883600	2.91022200	-3.36500800
C	2.26156300	3.96270600	-2.58952600

H	2.25268000	4.71569300	-3.38820000
H	2.80848500	4.39273200	-1.73981300
C	0.82224800	3.61428600	-2.16950100
H	0.31400000	4.50997200	-1.79230600
C	3.00292400	1.62320700	-1.95569800
H	3.57768700	1.99589900	-1.09981800
H	3.50695100	0.73653700	-2.34596200
C	0.04933100	3.04231200	-3.37219800
H	-0.00044900	3.78834600	-4.17562300
H	-0.98366200	2.80811200	-3.08042200
C	2.20343100	2.11019900	-4.29291100
H	2.70975800	1.20902200	-4.66420500
H	2.19539500	2.84162700	-5.11102000
C	2.19479400	0.21436500	1.52449100
C	1.63466400	0.64231500	4.39860300
H	1.94891200	0.78689500	5.44008500
H	0.53674900	0.66717600	4.38270100
C	4.23027700	0.37638600	3.02370900
H	5.32657400	0.34568200	3.01845700
C	1.74240000	1.59786600	2.05465500
H	2.20521400	2.38464300	1.45282000
H	0.65950900	1.71513600	1.97793300
C	2.13966500	-0.71873500	3.88126200
H	1.73347700	-1.52864000	4.49950300
C	3.74662300	0.16050300	1.56847700
H	4.10280600	-0.81670300	1.23277300
H	4.17471300	0.92590100	0.91055600
C	2.20487000	1.77062300	3.51845700
H	1.84231500	2.74158800	3.87665600
C	3.67871100	-0.75421700	3.91248400
H	4.04114000	-1.72772700	3.55572300
H	4.03548100	-0.62922800	4.94278700
C	1.64424900	-0.92066300	2.43344800
H	0.54790300	-0.91792500	2.42674500
H	1.97649300	-1.89509100	2.05240700
C	3.74389600	1.73575800	3.55716600
H	4.10124700	1.88231000	4.58459800
H	4.15482500	2.55222700	2.94862800
Au	-0.64948000	-1.08064100	0.00085800
N	0.27681300	-2.86379300	-1.08329400
P	1.50740600	-0.10831200	-0.21994200
C	-1.58228600	0.61511500	0.68546100
C	-2.26058400	1.36662600	-0.27406900
C	-1.70144400	0.93778400	2.04032500
C	-3.07528900	2.43834900	0.11044600
C	-2.50710600	2.00482900	2.42796100
C	-3.19936900	2.76376300	1.46828800

H	-3.59434400	3.00336400	-0.65480600
H	-2.61361400	2.26383400	3.47710600
H	-1.18574200	0.36654000	2.80417600
C	-2.36426800	-2.31208600	1.35950400
H	-1.73181000	-3.16458600	1.59095300
H	-2.55222800	-1.61018300	2.16682800
C	-3.09134900	-2.25131500	0.20410600
H	-2.95260000	-3.03961500	-0.53534400
H	-2.17683100	1.13835500	-1.33210700
C	-4.18260300	-1.26922400	-0.07367200
H	-4.01469200	-0.80114000	-1.05276100
H	-4.17956700	-0.47483600	0.67831800
C	-5.55865800	-1.97086200	-0.08959100
H	-5.55601800	-2.77563200	-0.83745200
H	-5.73286200	-2.44477300	0.88573100
O	-3.95378400	3.78071400	1.95378400
C	-4.68099800	4.59022800	1.02694300
H	-4.00640400	5.10242100	0.32941000
H	-5.40941800	3.99437000	0.46263100
H	-5.20827900	5.33111500	1.63066600
C	-6.69400600	-0.98591900	-0.39472800
H	-6.51209500	-0.51772100	-1.37236900
H	-6.67597300	-0.17496900	0.34662000
C	-8.06974000	-1.65722800	-0.39129000
H	-8.28751500	-2.10639400	0.58593600
H	-8.86457100	-0.93478100	-0.61168900
H	-8.12348100	-2.45419800	-1.14378200

$TS_{2\text{-}3}$

E (SMD/B3LYP-D3/BS1) = -2204.84213862

G (SMD/B3LYP-D3/BS1) = -2203.979282

E (SMD/B3LYP-D3/BS2//SMD/B3LYP-D3/BS1) = -2205.51558925

C	1.54409400	-1.98895400	-1.25505100
C	2.83353600	-2.33218400	-1.70972100
H	3.67608500	-1.69848200	-1.47320000
C	3.05917000	-3.48509000	-2.45422000
H	4.06496700	-3.72381600	-2.78559000
C	1.98902300	-4.32221400	-2.76641100
H	2.14541100	-5.22530900	-3.34848200
C	0.70503900	-3.99357400	-2.34111900
H	-0.11886100	-4.63836300	-2.62284400
C	0.47347600	-2.83686400	-1.58575300
C	-1.63503200	-3.65368000	-0.62835900
H	-1.60450800	-4.53151100	-1.27729800
H	-2.68036700	-3.38804900	-0.48057900
H	-1.16498300	-3.88672900	0.32854800

C	-1.61263100	-2.09857300	-2.52813700
H	-1.06174700	-1.27703800	-2.98612900
H	-2.63232000	-1.78383600	-2.31048300
H	-1.63676000	-2.94904800	-3.21710200
C	1.71660300	1.00793100	-1.37880300
C	0.90136400	0.82719100	-2.68837700
H	-0.17006600	0.78038800	-2.45298500
H	1.17562100	-0.11300200	-3.18066900
C	1.16833000	2.01086400	-3.64285900
H	0.59192200	1.85051600	-4.56237500
C	1.28204800	2.34020600	-0.71044700
H	0.22350000	2.30373100	-0.45315500
H	1.83863400	2.51078600	0.21194600
C	3.47629400	2.28070300	-2.67291500
H	4.54852900	2.31638000	-2.90136300
C	3.03972800	3.59432800	-1.99779500
H	3.24183100	4.44273900	-2.66466400
H	3.61702300	3.75680800	-1.07756600
C	1.53654400	3.52119700	-1.67149800
H	1.21576500	4.44269300	-1.16921000
C	3.22535500	1.08462000	-1.72359800
H	3.81985800	1.19517200	-0.81009300
H	3.55067400	0.17158200	-2.22847700
C	0.72821400	3.32280600	-2.96683600
H	0.89086000	4.16893700	-3.64703600
H	-0.34634000	3.28877400	-2.73891900
C	2.67274500	2.07482900	-3.97225400
H	2.98952400	1.14789800	-4.46924200
H	2.86750400	2.90113400	-4.66815300
C	2.24366300	-0.63724900	1.34994700
C	2.26474900	-0.26875500	4.28957500
H	2.72286800	-0.36850800	5.28196600
H	1.29625700	0.23249000	4.42692800
C	4.30194200	-1.50635400	2.55351500
H	5.26378600	-2.00873700	2.39343400
C	2.50825600	0.74909800	1.99060000
H	3.17678400	1.32882600	1.34597500
H	1.58324900	1.31666200	2.09391300
C	2.05423400	-1.66217800	3.66478700
H	1.40802700	-2.26821500	4.31199000
C	3.61257300	-1.34844800	1.17692400
H	3.47526900	-2.34266400	0.74312700
H	4.25692300	-0.77002000	0.50446600
C	3.17225100	0.57494800	3.37365200
H	3.31853200	1.57049300	3.81047300
C	3.41340300	-2.36160100	3.47641600
H	3.26652300	-3.35972000	3.04201100

H	3.90388600	-2.49728400	4.44902400
C	1.36285000	-1.50184600	2.29444900
H	0.38205000	-1.03164800	2.43495600
H	1.19350800	-2.48833100	1.84219000
C	4.53077700	-0.12556700	3.19388300
H	5.03094700	-0.24018200	4.16437000
H	5.18648500	0.48402500	2.55768000
Au	-1.12133900	-0.67660000	0.11096700
N	-0.92773700	-2.49169100	-1.25002600
P	1.26528800	-0.45961900	-0.27587300
C	-1.50364400	1.18205900	1.02612300
C	-2.17459800	2.08071200	0.17948400
C	-0.86545200	1.65353200	2.18384900
C	-2.12297700	3.45109100	0.42421800
C	-0.82396700	3.01496900	2.43909200
C	-1.42515900	3.92843100	1.54891100
H	-2.62592500	4.12903200	-0.25482300
H	-0.32001500	3.40008200	3.32009300
H	-0.40757700	0.96353000	2.88229500
C	-3.11313500	-0.16435000	1.83821300
H	-2.60006700	-0.39760600	2.76583100
H	-3.75608800	0.70800000	1.85262200
C	-3.25013900	-1.14561200	0.84237800
H	-2.97841400	-2.15563400	1.15254100
H	-2.72971700	1.72196400	-0.68218600
C	-4.33049200	-1.00810700	-0.20279600
H	-4.12627400	-1.67496900	-1.04528800
H	-4.34244400	0.02032000	-0.58527000
C	-5.71557800	-1.36094100	0.37905000
H	-5.68465500	-2.38049100	0.78831400
H	-5.93355900	-0.69003800	1.22172900
O	-1.29258100	5.22944100	1.87184100
C	-1.84370500	6.22039300	0.99468800
H	-1.40545200	6.14574800	-0.00769600
H	-2.93448000	6.13033000	0.93137000
H	-1.58232000	7.18226800	1.43811800
C	-6.83128500	-1.26073600	-0.66719300
H	-6.59722100	-1.92780300	-1.50890800
H	-6.85252400	-0.24038500	-1.07531600
C	-8.20632100	-1.61661700	-0.09538300
H	-8.47648900	-0.94635800	0.73044500
H	-8.98793200	-1.53752100	-0.86024200
H	-8.21921000	-2.64349100	0.29155700

3

E (SMD/B3LYP-D3/BS1) = -2204.87539397

G (SMD/B3LYP-D3/BS1) = -2204.008871

E (SMD/B3LYP-D3/BS2//SMD/B3LYP-D3/BS1) = -2205.54423247

C	1.43443000	-1.83600400	-1.29324700
C	2.71126200	-2.21543400	-1.75489800
H	3.57406900	-1.61932900	-1.49301700
C	2.89902300	-3.34556700	-2.54327100
H	3.89685800	-3.61001200	-2.87965800
C	1.79925100	-4.12693100	-2.89533200
H	1.92329900	-5.01195300	-3.51204000
C	0.52642500	-3.76464200	-2.46505700
H	-0.31821400	-4.36937700	-2.77359300
C	0.33546500	-2.62727300	-1.66732300
C	-1.77601700	-3.42105700	-0.71841200
H	-1.77052200	-4.27688700	-1.39707900
H	-2.81320400	-3.15228600	-0.53519200
H	-1.28566000	-3.69062700	0.21819100
C	-1.74907500	-1.81364000	-2.58064600
H	-1.16691900	-1.01138800	-3.03477000
H	-2.74885900	-1.45404200	-2.34098300
H	-1.82223700	-2.65075300	-3.28254600
C	1.80246300	1.11982600	-1.35062100
C	1.16363200	0.94006500	-2.75553200
H	0.07213200	0.85958200	-2.65639900
H	1.52174500	0.01637600	-3.22309600
C	1.51808300	2.14249400	-3.65741300
H	1.06446500	1.98292400	-4.64356200
C	1.24164600	2.43156600	-0.74679800
H	0.15349000	2.34695900	-0.67219400
H	1.62797500	2.59925000	0.26157500
C	3.68149900	2.44359300	-2.40409100
H	4.77264500	2.50710400	-2.49755300
C	3.13515300	3.73714300	-1.77112000
H	3.40241100	4.60229100	-2.39205900
H	3.58675200	3.89311500	-0.78191100
C	1.60378400	3.63455600	-1.64161400
H	1.21078200	4.54453400	-1.17301900
C	3.33865000	1.23693600	-1.50004100
H	3.80902600	1.36331400	-0.51960000
H	3.75085600	0.32988200	-1.95135100
C	0.97308900	3.44101200	-3.03249700
H	1.20813600	4.29714600	-3.67828500
H	-0.12164900	3.38821600	-2.94668700
C	3.05045000	2.23987800	-3.79569900
H	3.44533000	1.32526100	-4.25841700
H	3.31392100	3.07828600	-4.45376600
C	2.14856500	-0.59245200	1.34047800
C	2.09061200	-0.38776800	4.30481400
H	2.53173100	-0.53792200	5.29873000

H	1.11147300	0.08957600	4.45245800
C	4.18639600	-1.49768000	2.55400700
H	5.16133200	-1.97559300	2.39701800
C	2.35877800	0.75743700	2.07148800
H	3.01324500	1.40523900	1.48002500
H	1.40631600	1.27799500	2.19205400
C	1.91362700	-1.74481700	3.59558900
H	1.26045700	-2.39465500	4.19158300
C	3.52808500	-1.27911300	1.17219200
H	3.40855200	-2.25284000	0.68861100
H	4.18402500	-0.66934900	0.54128900
C	3.00009600	0.52115700	3.45589900
H	3.12104800	1.49282800	3.95105300
C	3.28774000	-2.41414300	3.40577800
H	3.16767700	-3.38871700	2.91341700
H	3.75384400	-2.59678400	4.38291000
C	1.25298000	-1.51196600	2.21983500
H	0.26819700	-1.04846800	2.36929500
H	1.09323700	-2.47255600	1.71179900
C	4.37470100	-0.14682400	3.26827700
H	4.85758000	-0.30000900	4.24235300
H	5.03168200	0.50518700	2.67667400
Au	-1.24738300	-0.47416100	0.08965500
N	-1.06144600	-2.25776000	-1.32409300
P	1.19402100	-0.31734900	-0.28482700
C	-2.18636600	1.13747000	1.39319000
C	-2.60967900	2.02922200	0.34101100
C	-1.18059300	1.63338100	2.29627700
C	-2.09533900	3.29825300	0.20570000
C	-0.66487500	2.89519400	2.16813600
C	-1.12581000	3.75186400	1.13266300
H	-2.44847600	3.94252500	-0.58951300
H	0.08644200	3.27545700	2.85180600
H	-0.85892600	0.99329400	3.11175900
C	-3.22861900	0.11434900	1.91806200
H	-2.88417200	-0.27031900	2.88051400
H	-4.19716100	0.60145800	2.06837300
C	-3.23123000	-0.96554400	0.86089000
H	-2.96115900	-1.94952000	1.25633900
H	-3.39597800	1.70988400	-0.33360600
C	-4.41018700	-1.00438400	-0.08467200
H	-4.19147600	-1.59800100	-0.97683800
H	-4.66825500	0.00634300	-0.42133200
C	-5.63879600	-1.62714000	0.61949500
H	-5.38664800	-2.64726100	0.94138100
H	-5.87297000	-1.05887100	1.52949000
O	-0.57744700	4.95761100	1.11007400

C	-1.02238500	5.94098500	0.14931100
H	-0.84832500	5.59250200	-0.87248800
H	-2.08265900	6.16478700	0.30221500
H	-0.41918800	6.82605400	0.34923700
C	-6.87319900	-1.66640700	-0.28994000
H	-6.63104500	-2.22662700	-1.20401700
H	-7.12096800	-0.64386400	-0.60712300
C	-8.08555500	-2.30103100	0.39740600
H	-8.36508200	-1.74322100	1.30018100
H	-8.95638500	-2.31783800	-0.26870400
H	-7.87333400	-3.33514300	0.69729100

TS_{3-4}
E (SMD/B3LYP-D3/BS1) = -2204.85898353
G (SMD/B3LYP-D3/BS1) = -2203.993493
E (SMD/B3LYP-D3/BS2//SMD/B3LYP-D3/BS1) = -2205.52829223

C	1.30237300	-2.17646700	-0.95206300
C	2.49826200	-2.50466800	-1.61934100
H	3.15950900	-1.71218000	-1.94588800
C	2.85753900	-3.82562200	-1.86689400
H	3.78582700	-4.04674200	-2.38461000
C	2.02462200	-4.85555500	-1.43121300
H	2.29842700	-5.89389100	-1.59181900
C	0.81842700	-4.55639200	-0.80285400
H	0.17339900	-5.37286100	-0.49768400
C	0.43615900	-3.22549500	-0.58537800
C	-1.03965700	-3.55840600	1.36984600
H	-0.88243600	-4.64152000	1.35550700
H	-2.04225100	-3.34192900	1.74245400
H	-0.29069100	-3.08744200	2.00717300
C	-1.93953700	-3.60606300	-0.92412900
H	-1.87537400	-3.12419000	-1.90018000
H	-2.93312400	-3.47230200	-0.50137000
H	-1.76097300	-4.67908900	-1.03099000
C	1.00049300	0.66743300	-1.99172800
C	0.33113800	-0.11947700	-3.15173700
H	-0.67781200	-0.43876000	-2.85138400
H	0.90429200	-1.02422600	-3.38021000
C	0.24648600	0.76609000	-4.41322000
H	-0.22303500	0.18345100	-5.21554200
C	0.14249700	1.93383300	-1.70420000
H	-0.86864400	1.63540900	-1.39527500
H	0.57649000	2.51082500	-0.88002500
C	2.32072500	1.99575300	-3.69644400
H	3.33567700	2.29343200	-3.98701200
C	1.47642500	3.24782600	-3.39145600
H	1.42240900	3.89129800	-4.27918500

H	1.94788000	3.83424800	-2.59116200
C	0.05881900	2.81585000	-2.96939300
H	-0.54722300	3.70022100	-2.73601100
C	2.41660600	1.12093500	-2.42678300
H	2.88601900	1.69968500	-1.62495400
H	3.05495400	0.25484900	-2.62837500
C	-0.59600800	2.01919400	-4.11376400
H	-0.66954000	2.64567300	-5.01207400
H	-1.61683000	1.72815400	-3.83870400
C	1.66790900	1.18747800	-4.83425300
H	2.27362600	0.29973600	-5.06132200
H	1.62253800	1.79404700	-5.74799100
C	2.21286200	-0.05116100	0.88840800
C	2.71267900	0.76697800	3.70478000
H	3.40818800	0.98174200	4.52717600
H	1.70514400	0.93767500	4.08720500
C	4.64019600	0.04240400	1.61724200
H	5.66935700	-0.11163900	1.26994900
C	2.02662600	1.41394800	1.35880500
H	2.22613200	2.10441500	0.53204500
H	0.98948000	1.57607700	1.68452700
C	2.88302800	-0.69425700	3.24950000
H	2.64752400	-1.37291300	4.07916900
C	3.68338800	-0.27854000	0.44940100
H	3.82793100	-1.32280100	0.15768200
H	3.93645400	0.34588200	-0.40993100
C	2.99808300	1.71388000	2.52430600
H	2.84900900	2.75432400	2.84019500
C	4.33682000	-0.90904400	2.78978400
H	4.48967900	-1.95058500	2.47573300
H	5.02338100	-0.71478900	3.62419900
C	1.91167400	-0.99563600	2.08366000
H	0.87808500	-0.85265300	2.42141500
H	2.01900400	-2.04259000	1.77366900
C	4.44927900	1.50858400	2.05044900
H	5.14508700	1.75567200	2.86286300
H	4.67547400	2.18011400	1.21083800
Au	-1.33361300	-0.77410200	0.22494000
N	-0.91988200	-2.99978900	-0.01216200
P	0.94829200	-0.44193800	-0.46174400
C	-2.70740700	0.05219600	2.61806900
C	-2.66939600	1.45431400	2.46397400
C	-1.78801600	-0.54006300	3.51297400
C	-1.77967400	2.24365400	3.18424400
C	-0.91490200	0.23717700	4.25685600
C	-0.90726900	1.63729300	4.10478400
H	-1.79501700	3.31905100	3.05500300

H	-0.23599600	-0.21423000	4.97248900
H	-1.79971700	-1.61667400	3.65271600
C	-3.79327400	-0.79936800	1.96072000
H	-3.81267800	-1.78508500	2.43262900
H	-4.78336600	-0.34405500	2.10464500
C	-3.51983400	-0.94852200	0.48780500
H	-3.76070300	-1.92073600	0.06544900
H	-3.38107700	1.94088600	1.80506500
C	-3.69430100	0.22141900	-0.42337700
H	-2.75788100	0.85995600	-0.41621100
H	-4.41833900	0.92495700	0.01757700
C	-4.01267000	-0.11669700	-1.88453800
H	-3.18334900	-0.69784000	-2.30966800
H	-4.90052000	-0.76093300	-1.91677000
O	-0.03324300	2.30824000	4.88298400
C	0.11123200	3.72319100	4.70874700
H	0.39534500	3.96784000	3.67870000
H	-0.81290400	4.24967300	4.97355200
H	0.90983600	4.02120700	5.38938800
C	-4.25035400	1.14191100	-2.72446800
H	-3.39755800	1.82367200	-2.60005700
H	-5.12916800	1.67336200	-2.33464100
C	-4.44800500	0.82838000	-4.20892700
H	-5.30982200	0.16660000	-4.36119800
H	-4.62011400	1.74265300	-4.78814700
H	-3.56542700	0.32802000	-4.62754600

5

E (SMD/B3LYP-D3/BS1) = -2204.88725336

G (SMD/B3LYP-D3/BS1) = -2204.020721

E (SMD/B3LYP-D3/BS2//SMD/B3LYP-D3/BS1) = -2205.56262545

C	1.35637800	1.13945800	1.98639100
C	2.02548700	2.31842200	2.36721700
H	2.24759600	3.07951000	1.63041900
C	2.42562200	2.51408200	3.68519300
H	2.93855900	3.42954300	3.96283500
C	2.17335800	1.52522900	4.63687100
H	2.48437400	1.66307500	5.66785900
C	1.52621600	0.34762600	4.26935800
H	1.34862300	-0.41240700	5.02157100
C	1.11574100	0.14467100	2.94729400
C	-0.81509400	-1.24875500	3.40010500
H	-0.59349900	-1.24981400	4.47148100
H	-1.30697800	-2.18470300	3.14255100
H	-1.46613600	-0.40711200	3.16348000
C	1.34516900	-2.28459400	2.88037700
H	1.58022500	-2.34536300	3.94736800

H	2.26678800	-2.16193800	2.31037700
H	0.84406600	-3.20389800	2.57738800
C	2.57216900	0.61269600	-0.70504500
C	3.56937400	-0.12470500	0.22900800
H	3.14610900	-1.08810600	0.54221200
H	3.75469000	0.46283300	1.13357000
C	4.90516700	-0.35474300	-0.51213400
H	5.59107500	-0.86979600	0.17131200
C	2.34273900	-0.27279500	-1.95752200
H	1.94424500	-1.24140500	-1.64001600
H	1.61043800	0.18065700	-2.63221300
C	4.51841800	1.73009600	-1.86512800
H	4.92977300	2.70436000	-2.15536600
C	4.26561500	0.87259600	-3.11967000
H	5.20538100	0.72203600	-3.66609200
H	3.57274800	1.38991500	-3.79690800
C	3.67927300	-0.48856300	-2.69896600
H	3.48255500	-1.10289100	-3.58576400
C	3.18079900	1.97568200	-1.13033400
H	2.49414900	2.50961700	-1.79353900
H	3.35834400	2.60698700	-0.25301100
C	4.66589500	-1.21479200	-1.76626800
H	5.61551100	-1.39218100	-2.28702600
H	4.26357700	-2.19622400	-1.48085000
C	5.49959900	1.00606600	-0.92393200
H	5.69074100	1.62004200	-0.03362300
H	6.46187600	0.85534100	-1.42967800
C	-0.26834000	2.11791600	-0.41085000
C	-2.85086100	2.66774500	-1.74815500
H	-3.62144400	3.34735600	-2.13340500
H	-3.19142500	1.64399800	-1.94771000
C	-0.87086600	4.56264000	-0.65946800
H	-0.51922100	5.58033000	-0.45090000
C	-0.44911200	1.93172900	-1.93860000
H	0.49791100	2.10738500	-2.45848400
H	-0.76320500	0.90425000	-2.16259800
C	-2.67904200	2.87526400	-0.23196600
H	-3.63020800	2.67592800	0.27189100
C	0.18457700	3.57450500	-0.11091400
H	0.27324600	3.72047600	0.96959900
H	1.15854300	3.78379900	-0.56246900
C	-1.50954300	2.92659300	-2.45821800
H	-1.62152000	2.77444200	-3.53868500
C	-2.21645400	4.31644900	0.04751800
H	-2.11027100	4.47778300	1.12878100
H	-2.96848600	5.02803600	-0.31700600
C	-1.62797900	1.87663400	0.29912300

H	-1.97870800	0.85656600	0.10882400
H	-1.51073400	1.99093200	1.38458800
C	-1.03583400	4.36565700	-2.17749300
H	-1.76719900	5.08383900	-2.56997300
H	-0.08311300	4.55609400	-2.68986900
Au	-0.13868000	-1.23259500	0.41460900
N	0.43947100	-1.12715900	2.59757500
P	0.94506700	0.83307200	0.23777100
C	-3.52464300	-2.14093100	0.53212700
C	-3.98985700	-2.06017700	-0.78657400
C	-3.83847900	-1.08042500	1.39940100
C	-4.70525600	-0.95014300	-1.24784400
C	-4.55865700	0.02553100	0.96318300
C	-4.98826300	0.10658600	-0.37051400
H	-5.05219300	-0.93049700	-2.27434200
H	-4.80580200	0.83584500	1.64203000
H	-3.53390200	-1.13224200	2.44003400
C	-2.74331600	-3.33261100	1.04589100
H	-2.75535500	-3.32943700	2.13899700
H	-3.24405500	-4.26552100	0.74564300
C	-1.30025800	-3.48110400	0.58062900
H	-0.63087400	-3.97574100	1.28410000
H	-3.81838300	-2.88602400	-1.47190200
C	-0.89579900	-3.39634300	-0.72935700
H	-1.62647400	-3.06522400	-1.46488000
H	-0.44198400	-1.08326400	-1.11812200
C	0.38854700	-3.94761400	-1.29863600
H	1.23991000	-3.70250800	-0.65066500
H	0.30159700	-5.04457600	-1.27895200
O	-5.66879800	1.23465900	-0.70705200
C	-6.19438300	1.34271200	-2.03050200
H	-5.39827200	1.31823600	-2.78388200
H	-6.91859000	0.54456800	-2.23628100
H	-6.69893900	2.30985800	-2.07076700
C	0.64077900	-3.49093700	-2.74204500
H	0.57390200	-2.39684300	-2.79279200
H	-0.16491000	-3.87950000	-3.37883900
C	1.99741900	-3.95326200	-3.27395300
H	2.14679500	-3.63013700	-4.31030200
H	2.81763900	-3.54217800	-2.67288600
H	2.08051000	-5.04696000	-3.24800100

$TS_{2\text{-}6}$

E (SMD/B3LYP-D3/BS1) = -2204.85277027 6

G (SMD/B3LYP-D3/BS1) = -2203.988638

E (SMD/B3LYP-D3/BS2//SMD/B3LYP-D3/BS1) = -2205.52523651

C	1.47066500	-2.03508600	-1.30786800

C	2.68966800	-2.40019500	-1.91226800
H	3.56229200	-1.77329000	-1.78803200
C	2.80527100	-3.56867700	-2.65833000
H	3.75526000	-3.82481900	-3.11725100
C	1.69908200	-4.40641700	-2.80086300
H	1.77271500	-5.32457500	-3.37568700
C	0.49254100	-4.07583100	-2.18963000
H	-0.34669400	-4.75470300	-2.29025900
C	0.36924800	-2.89677100	-1.44307900
C	-1.17875200	-3.69012300	0.25375800
H	-1.25815800	-4.66990900	-0.22687100
H	-2.11471600	-3.47629500	0.77046700
H	-0.35531300	-3.69937800	0.96817300
C	-2.03696000	-2.61253700	-1.77428100
H	-1.86350900	-1.80197400	-2.48475600
H	-2.98069200	-2.45563300	-1.25308700
H	-2.09281800	-3.56397200	-2.30870400
C	1.77370100	0.97293400	-1.33245500
C	0.63499400	1.10912800	-2.38175900
H	-0.33658900	1.16503200	-1.88028800
H	0.62064700	0.22248200	-3.02923200
C	0.85023100	2.37952500	-3.23390600
H	0.02891500	2.45504600	-3.95763800
C	1.79768500	2.23147600	-0.42452000
H	0.86740000	2.31403100	0.14446600
H	2.61832200	2.15528300	0.29663500
C	3.33275100	2.15625100	-2.94369800
H	4.29584200	2.06510100	-3.46094100
C	3.34703600	3.39371700	-2.02624300
H	3.52116500	4.29943100	-2.62190000
H	4.16827200	3.31696000	-1.30073900
C	1.99839800	3.49528700	-1.28931400
H	1.99700600	4.36971400	-0.62643600
C	3.11698800	0.87567000	-2.10337000
H	3.95633200	0.74197400	-1.41651800
H	3.09734200	0.01825200	-2.78190600
C	0.85507700	3.61747900	-2.31568400
H	0.98902800	4.52678800	-2.91624000
H	-0.10694200	3.70607300	-1.79736500
C	2.19640000	2.28415800	-3.97520500
H	2.19808100	1.41568700	-4.64785600
H	2.34749300	3.17801600	-4.59455600
C	2.45747400	-0.82766100	1.23464900
C	2.65220800	-1.71187700	4.07633600
H	3.23462200	-1.91552000	4.98426500
H	1.60265100	-1.94669500	4.30317700
C	4.77552600	-0.79223900	2.24553900

H	5.82644900	-0.56066600	2.03300500
C	1.94356200	0.03403000	2.41476600
H	1.96275900	1.09938700	2.16560500
H	0.90235700	-0.23607500	2.61763200
C	3.15464200	-2.59395900	2.91833400
H	3.04418800	-3.65358000	3.17996100
C	3.94899100	-0.50497300	0.97150300
H	4.33283400	-1.10895900	0.14278400
H	4.06474300	0.54839400	0.69965000
C	2.78823000	-0.22991600	3.67923000
H	2.41190800	0.41009200	4.48716500
C	4.63527600	-2.27686900	2.63191700
H	5.00685700	-2.91462500	1.81843800
H	5.24302500	-2.49330400	3.52013400
C	2.32044000	-2.31892000	1.64771300
H	1.26548800	-2.55593800	1.84027800
H	2.66408400	-2.97467700	0.84167500
C	4.26644500	0.09815100	3.39535900
H	4.86755200	-0.07234000	4.29794300
H	4.37337700	1.15787600	3.12669200
Au	-1.01854200	-0.61595200	0.26465500
N	-0.92577900	-2.62454700	-0.76899400
P	1.34126600	-0.52876100	-0.26990100
C	-1.38838300	1.29797500	1.03762700
C	-1.85234600	2.23937900	0.09375000
C	-0.91879800	1.76104200	2.28788100
C	-1.80937900	3.59972900	0.35807800
C	-0.87949300	3.11596000	2.56203600
C	-1.30322000	4.05135500	1.59445000
H	-2.17164700	4.29788500	-0.38629400
H	-0.52221400	3.48167800	3.51941600
H	-0.60691800	1.05857500	3.05023000
C	-3.25424400	0.31549200	1.71085100
H	-2.89820800	0.09887500	2.71670000
C	-3.12229300	-0.75029800	0.75750400
H	-3.15229100	-1.75097700	1.19480100
H	-2.26560400	1.90417800	-0.85193500
O	-1.20544100	5.34118200	1.95035900
C	-1.60243800	6.35797700	1.01686100
H	-1.00836200	6.29929900	0.09797300
H	-2.66942900	6.27792500	0.78055100
H	-1.40828500	7.30603900	1.51991800
C	-4.27408200	1.39591300	1.62255500
H	-3.91287300	2.30005000	2.12525700
H	-4.49433600	1.63628200	0.57834300
C	-5.56581100	0.90268700	2.32651200
H	-5.91882000	-0.01605800	1.84006400

H	-5.34255000	0.64857300	3.37102600
C	-6.66749500	1.97035400	2.27805500
H	-6.87359600	2.22838300	1.23038200
H	-6.30212000	2.88681200	2.76089600
C	-7.95656300	1.50337400	2.95904400
H	-8.73001000	2.27915000	2.91317200
H	-8.35597900	0.60297700	2.47545100
H	-7.78224500	1.26591100	4.01599600
H	-3.69997700	-0.63317300	-0.16074700

6

E (SMD/B3LYP-D3/BS1) = -2204.87736620

G (SMD/B3LYP-D3/BS1) = -2204.011333

E (SMD/B3LYP-D3/BS2//SMD/B3LYP-D3/BS1) = -2205.54656586

C	1.36232200	-1.90472800	-1.21232300
C	2.58613300	-2.36194300	-1.73970000
H	3.49461200	-1.80968900	-1.53776900
C	2.65984600	-3.51747000	-2.51014900
H	3.61675900	-3.84448000	-2.90506900
C	1.49894200	-4.24741000	-2.76602300
H	1.53500700	-5.15037000	-3.36786400
C	0.28295500	-3.82882000	-2.23332000
H	-0.60231300	-4.42629400	-2.41955400
C	0.20879100	-2.66666600	-1.45278100
C	-1.46969500	-3.38899300	0.14390900
H	-1.52267700	-4.36171700	-0.35380100
H	-2.44336100	-3.16349900	0.57600500
H	-0.71034200	-3.41822600	0.92657600
C	-2.15891700	-2.21335100	-1.91069000
H	-1.87808200	-1.41678600	-2.60130400
H	-3.11417000	-1.98311700	-1.44084200
H	-2.25917700	-3.15590500	-2.45415200
C	1.78414800	1.04116700	-1.36548100
C	0.63384100	1.20279300	-2.40020200
H	-0.32167000	1.35884800	-1.88109100
H	0.53858200	0.28716100	-2.99813800
C	0.91618300	2.40749200	-3.32488100
H	0.09142300	2.49643600	-4.04310400
C	1.89102500	2.33790500	-0.52771200
H	0.96560000	2.48673900	0.03572600
H	2.70640600	2.25362600	0.19821500
C	3.38603600	2.04996400	-3.05049500
H	4.33490100	1.87923200	-3.57376800
C	3.48492600	3.32818800	-2.19536300
H	3.70438600	4.19237400	-2.83602600
H	4.30930400	3.23791800	-1.47505600
C	2.15388000	3.54665400	-1.45111600

H	2.21269600	4.44949500	-0.83044900
C	3.10632700	0.82807900	-2.14541500
H	3.94319400	0.67939200	-1.45732400
H	3.02989700	-0.06135800	-2.77746500
C	1.01029300	3.68841900	-2.47427800
H	1.19369800	4.55638300	-3.12112300
H	0.05931200	3.86422100	-1.95735700
C	2.24406500	2.19169700	-4.07413200
H	2.18435600	1.29008800	-4.69869800
H	2.43816500	3.04062200	-4.74277200
C	2.38919300	-0.64187800	1.29239700
C	2.50960900	-1.38241300	4.17924300
H	3.07048900	-1.57211100	5.10366800
H	1.44596700	-1.54339900	4.40511100
C	4.69837000	-0.68368200	2.32620000
H	5.76286600	-0.52503900	2.11389300
C	1.92236200	0.31241400	2.42138300
H	2.01898300	1.35830900	2.11403100
H	0.86246000	0.13152200	2.62393000
C	2.96973300	-2.35274500	3.07522900
H	2.79795900	-3.38871500	3.39270600
C	3.89913300	-0.42009000	1.02973500
H	4.25430800	-1.09337000	0.24281500
H	4.07918900	0.60635400	0.69523900
C	2.73679800	0.06613300	3.70783400
H	2.39162700	0.76844800	4.47722900
C	4.46897800	-2.13529300	2.78963600
H	4.81098400	-2.83613200	2.01605500
H	5.05546200	-2.33760700	3.69537300
C	2.16549600	-2.09953200	1.78118700
H	1.09541900	-2.27376700	1.96404900
H	2.48511800	-2.81236500	1.01421100
C	4.23387800	0.29298500	3.42366800
H	4.81783800	0.13510000	4.33977900
H	4.40428500	1.32960500	3.10230000
Au	-1.13550800	-0.35581400	0.26428700
N	-1.10648400	-2.32535200	-0.84965100
P	1.27198500	-0.36842200	-0.21335900
C	-2.02470000	1.42766000	1.44307900
C	-2.17298500	2.37546900	0.36307700
C	-1.11618900	1.78937600	2.50158900
C	-1.49811400	3.57188500	0.34620800
C	-0.43178600	2.97426300	2.48703900
C	-0.62243300	3.88950800	1.41587300
H	-1.64842000	4.26794500	-0.46837300
H	0.24355300	3.25163300	3.28914000
H	-1.01960500	1.11505000	3.34638300

C	-3.25077400	0.53404100	1.79165100
H	-3.00233700	0.00116700	2.71699600
C	-3.22820900	-0.44406600	0.64358500
H	-3.47961800	-1.47615200	0.88483900
H	-2.87849900	2.15364500	-0.43080700
O	0.06919700	5.01419700	1.50275700
C	-0.08288300	6.04535200	0.50043900
H	0.19923500	5.66781600	-0.48484200
H	-1.11253400	6.41471500	0.49202100
H	0.59728400	6.83966000	0.80646100
C	-4.55303000	1.31497700	2.00824800
H	-4.38418900	2.06796800	2.79105800
H	-4.80869900	1.85793900	1.08880900
C	-5.71522800	0.39673200	2.40508700
H	-5.87343800	-0.35373300	1.61681700
H	-5.44264500	-0.16093900	3.31332600
C	-7.02578700	1.15416100	2.64874900
H	-7.29232000	1.71648400	1.74255600
H	-6.87010900	1.90011300	3.44087500
C	-8.18051500	0.22571300	3.03554100
H	-9.10667400	0.78750400	3.20571300
H	-8.37643800	-0.51068500	2.24540400
H	-7.95123800	-0.32759600	3.95534800
H	-3.72608800	-0.08932700	-0.26343300

TS_{6-7}

E (SMD/B3LYP-D3/BS1) = -2204.84456035

G (SMD/B3LYP-D3/BS1) = -2203.980442

E (SMD/B3LYP-D3/BS2//SMD/B3LYP-D3/BS1) = -2205.51556692

C	1.30248100	-1.94791500	-1.27394200
C	2.51501200	-2.50742200	-1.71685000
H	3.45108300	-2.04762000	-1.42292500
C	2.53829600	-3.64277500	-2.52116500
H	3.48691800	-4.05496700	-2.85049600
C	1.33677300	-4.24347200	-2.89485600
H	1.33402200	-5.13045200	-3.52089500
C	0.12331200	-3.71611500	-2.45782100
H	-0.79546500	-4.21571900	-2.74186300
C	0.09777100	-2.57314300	-1.64992000
C	-1.81344200	-3.14266100	-0.23302400
H	-1.94607900	-4.08643500	-0.77035000
H	-2.77838500	-2.79828100	0.13827300
H	-1.11386000	-3.27917700	0.59193000
C	-2.18139300	-1.87592200	-2.32690500
H	-1.75751700	-1.10397000	-2.96948700
H	-3.14681500	-1.55786900	-1.93561800
H	-2.31740800	-2.80023800	-2.89375400

C	1.90614300	0.98722300	-1.29970400
C	0.97966400	1.05008300	-2.54929900
H	-0.07298500	1.12311600	-2.23849500
H	1.08594700	0.13285500	-3.14110200
C	1.34261400	2.27708500	-3.41301000
H	0.67612600	2.29728300	-4.28432800
C	1.72755900	2.29101100	-0.48260100
H	0.69249100	2.37402000	-0.13130200
H	2.37222900	2.27417600	0.40273000
C	3.72919200	2.10946500	-2.64221400
H	4.77402400	2.01564000	-2.96260800
C	3.55286700	3.39458800	-1.80825700
H	3.83287300	4.27066500	-2.40771600
H	4.21772700	3.37126700	-0.93407600
C	2.08564000	3.51401400	-1.35157700
H	1.94698300	4.41941600	-0.74843700
C	3.37174900	0.87322000	-1.78344900
H	4.05556500	0.81027200	-0.93270700
H	3.50193800	-0.03316000	-2.38453600
C	1.15875400	3.56116400	-2.58084700
H	1.39131000	4.44201900	-3.19336900
H	0.11357800	3.65505000	-2.25703100
C	2.80798500	2.16257600	-3.87570100
H	2.94128400	1.25878700	-4.48551200
H	3.06990500	3.02325200	-4.50484800
C	2.14523100	-0.79057000	1.38980500
C	1.75274600	-1.51421100	4.24944000
H	2.14029300	-1.75266200	5.24823600
H	0.65576800	-1.55995800	4.30603900
C	4.25812600	-1.06685200	2.74623900
H	5.35286600	-1.02308100	2.69544500
C	1.62734400	0.23317100	2.43895000
H	1.91945200	1.25026300	2.15847500
H	0.52994700	0.21004100	2.47305600
C	2.27154600	-2.53884300	3.22297500
H	1.94683000	-3.54806400	3.50485700
C	3.69051200	-0.72189200	1.34966600
H	4.08880200	-1.41759300	0.60251900
H	4.00959300	0.28743100	1.06900200
C	2.20262400	-0.10170500	3.83175700
H	1.82662300	0.63800300	4.54947300
C	3.81081700	-2.48270800	3.15875800
H	4.18400700	-3.22214700	2.43732900
H	4.23576800	-2.73884500	4.13791900
C	1.69748500	-2.21267600	1.82893500
H	0.59939900	-2.26826400	1.85407100
H	2.04664300	-2.95880500	1.10689400

C	3.74203900	-0.04207500	3.77553700
H	4.16089600	-0.26154900	4.76618800
H	4.06987400	0.96909900	3.49875000
Au	-1.09921600	-0.25570600	-0.02050300
N	-1.24955700	-2.11579900	-1.17581900
P	1.27651500	-0.43842800	-0.24128700
C	-2.21612300	2.26274200	1.09237000
C	-2.53580500	3.06704100	-0.01668700
C	-1.21017600	2.72423800	1.97376500
C	-1.87423300	4.27112900	-0.25282000
C	-0.55297200	3.92183000	1.75852800
C	-0.86150100	4.69777600	0.62444700
H	-2.13933600	4.85866300	-1.12349000
H	0.22662900	4.26620600	2.43036900
H	-0.94737700	2.12160500	2.83891300
C	-2.89480500	0.92483300	1.41450100
H	-2.25432100	0.33728500	2.10070500
C	-3.18589600	0.15387300	0.15896100
H	-3.71866000	-0.78809800	0.28196100
H	-3.31324300	2.76530200	-0.71109600
O	-0.11862800	5.81247600	0.46058300
C	-0.34244300	6.63254900	-0.69307300
H	-0.16344100	6.07067600	-1.61659800
H	-1.35985200	7.04030800	-0.69510800
H	0.37789400	7.44824300	-0.61600500
C	-4.20735900	1.17244900	2.25240600
H	-3.91134000	1.84837000	3.06616300
H	-4.90806000	1.71836700	1.60839400
C	-4.86074200	-0.08336300	2.82755200
H	-5.20196600	-0.74014300	2.01655500
H	-4.11392700	-0.65173000	3.40214800
C	-6.05495100	0.24710900	3.73407000
H	-6.78831600	0.83358900	3.16316900
H	-5.71692300	0.89112600	4.55767200
C	-6.72874700	-1.00513300	4.30163800
H	-7.57826800	-0.74365200	4.94353000
H	-7.10390100	-1.65092300	3.49724700
H	-6.02510700	-1.59572500	4.90225900
H	-3.50765300	0.70416500	-0.72304000

8

E (SMD/B3LYP-D3/BS1) = -2204.89538743

G (SMD/B3LYP-D3/BS1) = -2204.033113

E (SMD/B3LYP-D3/BS2//SMD/B3LYP-D3/BS1) = -2205.57123213

C	0.95654800	-1.79906000	-1.23843200
C	2.18211300	-1.95148200	-1.91332100
H	3.00127600	-1.27596600	-1.70048700

C	2.36330200	-2.97195700	-2.84147100
H	3.31491500	-3.07448800	-3.35349300
C	1.31857200	-3.86000400	-3.10019400
H	1.44513900	-4.66129600	-3.82180700
C	0.10548400	-3.73176700	-2.42647400
H	-0.68767800	-4.44141800	-2.63295900
C	-0.08540300	-2.70942100	-1.49034400
C	-1.59571500	-3.87904500	0.03161800
H	-1.63656400	-4.75888100	-0.61726700
H	-2.54162900	-3.78779400	0.56802900
H	-0.77507400	-3.98534500	0.74384200
C	-2.49843100	-2.46598800	-1.75697300
H	-2.35166800	-1.53393100	-2.30605700
H	-3.44270100	-2.43255700	-1.21460500
H	-2.53040400	-3.30299900	-2.46045000
C	0.81444300	1.20782600	-0.80035800
C	-0.42870200	1.32454000	-1.72766400
H	-1.34944700	1.18926000	-1.14654200
H	-0.39904000	0.54123400	-2.49553000
C	-0.45949600	2.71860000	-2.39072100
H	-1.34661700	2.77317000	-3.03416900
C	0.74063600	2.30956200	0.28772200
H	-0.15573200	2.17103400	0.90623400
H	1.61420300	2.25073500	0.94505900
C	2.04910900	2.81364900	-2.31449400
H	2.96413600	2.93968500	-2.90574600
C	1.97939300	3.89569000	-1.22040700
H	1.97696200	4.89233800	-1.68023700
H	2.86445800	3.83713700	-0.57281300
C	0.69951300	3.69874000	-0.38479800
H	0.64701800	4.46031400	0.40266900
C	2.08874000	1.41004400	-1.66591300
H	2.99214400	1.30885700	-1.05731500
H	2.12718100	0.65355600	-2.45575800
C	-0.54021100	3.79872200	-1.29396300
H	-0.58966400	4.79484100	-1.75245000
H	-1.45575200	3.66624900	-0.70131800
C	0.81531600	2.92189200	-3.23036000
H	0.86817900	2.16741600	-4.02663100
H	0.79220500	3.90697600	-3.71390600
C	1.90376800	-0.82729000	1.46518300
C	2.29501900	-2.03122500	4.15738200
H	2.92735500	-2.28820000	5.01678200
H	1.28211400	-2.39507700	4.37776100
C	4.24558800	-0.67814800	2.40335800
H	5.25911500	-0.31689600	2.19125200
C	1.35530200	-0.16425000	2.75976000

H	1.28638100	0.92123600	2.64162500
H	0.34733300	-0.53943600	2.96767800
C	2.83481400	-2.70747900	2.88403500
H	2.83987500	-3.79678500	3.01174100
C	3.34380700	-0.31626500	1.20140200
H	3.75120800	-0.76624700	0.29031200
H	3.33749500	0.76973100	1.06424500
C	2.27451200	-0.50515600	3.95274200
H	1.87038300	-0.01418800	4.84662200
C	4.26422600	-2.20688600	2.59612300
H	4.66084100	-2.69601100	1.69623400
H	4.92676700	-2.47061700	3.43043300
C	1.92531400	-2.36541200	1.68384200
H	0.90420600	-2.72666200	1.87080800
H	2.29761800	-2.87555100	0.78950800
C	3.70202300	0.00223600	3.67386000
H	4.35255200	-0.22371000	4.52851500
H	3.69760400	1.09297300	3.54525200
Au	-1.45398900	-0.90450800	0.66294700
N	-1.38408700	-2.63244900	-0.77371300
P	0.74436600	-0.48799400	0.01399900
C	-4.08126400	1.31726900	1.35166500
C	-4.10926900	1.43274900	-0.05744800
C	-4.36701600	2.48419300	2.10300900
C	-4.38463100	2.63365300	-0.69255300
C	-4.66313100	3.68443100	1.48409200
C	-4.66313000	3.77968300	0.07869300
H	-4.36876000	2.68050000	-1.77455400
H	-4.89279000	4.57176900	2.06514000
H	-4.37670300	2.45086400	3.18629900
C	-3.70357800	0.06681300	2.00482400
H	-1.30598900	0.32285400	1.62262200
C	-3.62688100	-1.14785300	1.29631100
H	-3.58209300	-2.06965000	1.87010800
H	-3.86860700	0.57832900	-0.68019200
O	-4.92951500	4.99476000	-0.42927400
C	-4.92286200	5.17094200	-1.85319500
H	-3.93531000	4.94454100	-2.27203400
H	-5.68393000	4.54404800	-2.33141100
H	-5.15786100	6.22320200	-2.01839200
C	-3.35533100	0.09875000	3.47653600
H	-2.72209600	0.97431600	3.66330200
H	-4.29425200	0.29910700	4.01524100
C	-2.68679500	-1.14476000	4.07368700
H	-3.37524500	-1.99908100	4.04374900
H	-1.81456000	-1.41954200	3.46332400
C	-2.23295100	-0.91103900	5.51936300

H	-3.09693700	-0.61256800	6.12881900
H	-1.52841300	-0.06751800	5.54468800
C	-1.57300800	-2.14919500	6.13113700
H	-1.25480400	-1.96191600	7.16324900
H	-2.26486500	-3.00096200	6.14353900
H	-0.68512500	-2.44797500	5.55875300
H	-4.16274900	-1.23073600	0.35510800

$TS_{2\text{-}9}$

E (SMD/B3LYP-D3/BS1) = -3166.45511558

G (SMD/B3LYP-D3/BS1) = -3165.581968

E (SMD/B3LYP-D3/BS2//SMD/B3LYP-D3/BS1) = -3167.48241765

C	1.74285900	-1.53764300	-1.54024500
C	2.81249700	-1.40257100	-2.44804400
H	3.05496300	-0.43235500	-2.85720800
C	3.57951400	-2.49608000	-2.83570000
H	4.39389000	-2.36085100	-3.54092800
C	3.29423600	-3.75684800	-2.31187100
H	3.88323000	-4.62204200	-2.60123200
C	2.24646100	-3.91022800	-1.40900500
H	2.04234000	-4.89600200	-1.00768000
C	1.46484300	-2.81271200	-1.02349900
C	0.95584600	-3.45629800	1.25203000
H	1.54269800	-4.37326100	1.13760700
H	0.15729200	-3.64194400	1.96919600
H	1.59800400	-2.65445800	1.62032200
C	-0.54678100	-4.10441900	-0.58595500
H	-0.85181000	-3.82961700	-1.59423800
H	-1.42172600	-4.18115900	0.05647600
H	-0.04498500	-5.07627400	-0.60735700
Au	-0.91388300	-1.19328700	0.37369200
N	0.36491300	-3.04021000	-0.05903200
P	0.71183800	-0.09368800	-1.05841100
C	-2.04838700	0.44374300	0.90105600
C	-3.23314300	0.74132500	0.22617300
C	-1.67289400	1.22809300	1.99813700
C	-4.01861700	1.83793700	0.60189300
C	-2.44825300	2.32225000	2.38031400
C	-3.62260000	2.64039700	1.68099300
H	-4.92902700	2.04578800	0.05162700
H	-2.15460100	2.94282300	3.22201400
H	-0.77036500	1.00759700	2.55858800
C	-2.19615200	-2.24379600	1.80664100
H	-1.78348400	-3.24701400	1.90799100
H	-2.04974900	-1.65597600	2.71561100
C	-3.58055800	-2.24467700	1.37489300
H	-4.14309700	-1.32766500	1.54560000

H	-3.56727200	0.12066300	-0.60012300
C	-4.20990500	-3.23171100	0.64056700
C	-5.70621400	-3.23511100	0.39000200
H	-6.07508400	-2.20118300	0.35777000
H	-5.90528000	-3.67642600	-0.59339900
O	-4.30465600	3.72961900	2.12976200
C	-5.51546900	4.08902900	1.46529600
H	-5.33812900	4.33298200	0.40986900
H	-6.26379700	3.28881200	1.53371600
H	-5.88720300	4.97572200	1.98279700
C	-6.47397600	-4.02696100	1.46232000
H	-6.28551200	-3.57193100	2.44450600
H	-6.07441300	-5.04937300	1.50962700
C	-7.97844700	-4.07177400	1.18472300
H	-8.18650400	-4.54879900	0.21859900
H	-8.50906800	-4.63765400	1.95970200
H	-8.40489100	-3.06089500	1.15584400
H	-3.71082100	-4.20064600	0.56398100
H	-3.69778500	-2.64524500	-0.49614600
C	-0.01419800	0.63320200	-2.65224200
C	-0.83856400	2.83786200	-4.47473700
H	-1.32845700	3.20787400	-5.38530500
H	-0.42659600	3.70964600	-3.94795300
C	-1.32455900	-0.06676200	-4.70705100
H	-1.74243500	-0.93751500	-5.22547800
C	-1.16253000	1.61247900	-2.29763000
H	-1.89323700	1.10421500	-1.67174900
H	-0.78778400	2.46845700	-1.73053600
C	0.29182600	1.85950700	-4.84360100
H	1.03427700	2.36701300	-5.47232300
C	-0.62207900	-0.56568500	-3.42742600
H	0.16252300	-1.27962800	-3.70143600
H	-1.34151400	-1.09752100	-2.79703500
C	-1.85924800	2.11205100	-3.58038300
H	-2.65948900	2.80356300	-3.28673100
C	-0.29525300	0.65107300	-5.60157200
H	0.50820600	-0.04327100	-5.88337500
H	-0.77345300	0.99047500	-6.53010200
C	1.00184800	1.37514000	-3.55617700
H	1.42429500	2.23457700	-3.02419900
H	1.82780300	0.71774900	-3.83973700
C	-2.45556000	0.90929100	-4.33250500
H	-2.97642400	1.24711800	-5.23859400
H	-3.19484900	0.40027100	-3.70262000
C	1.83763000	1.05967700	-0.06346600
C	2.18718600	2.61530500	2.43711100
H	2.76869600	3.26179100	3.10745800

H	1.21741200	2.43371300	2.92040900
C	4.06870200	2.23748300	0.19629300
H	5.03379700	2.41097300	-0.29596400
C	1.13072200	2.42182800	0.15121500
H	1.00779400	2.92650600	-0.81257900
H	0.13422900	2.27770600	0.57785600
C	2.92887100	1.28159800	2.21969900
H	3.07421000	0.77414500	3.18174400
C	3.22206800	1.31797600	-0.71578400
H	3.75700300	0.37319000	-0.85114100
H	3.10554300	1.78130900	-1.70065800
C	1.97969200	3.31585600	1.08018900
H	1.44711800	4.26334500	1.22925800
C	4.29254000	1.54619400	1.55460100
H	4.83249900	0.60011900	1.41277900
H	4.91064900	2.18140000	2.20276500
C	2.08081800	0.36945000	1.30835500
H	1.12792500	0.14817500	1.80233600
H	2.59866600	-0.58535300	1.15274800
C	3.34387000	3.57881000	0.41495500
H	3.95472900	4.23527400	1.04877000
H	3.20056200	4.09277500	-0.54525000
S	-3.41878200	-3.34518800	-2.75049900
O	-3.84807700	-4.63352700	-2.19387500
O	-3.28539600	-2.25919400	-1.65999400
O	-2.28725600	-3.31837000	-3.68152500
C	-4.87533800	-2.69615900	-3.71080200
F	-5.90675600	-2.48431300	-2.88364100
F	-5.23102700	-3.58964200	-4.63729000
F	-4.55741000	-1.54427300	-4.31250000

$TS_{2\text{-}9}^{py}$

E (SMD/B3LYP-D3/BS1) = -2453.16107806

G (SMD/B3LYP-D3/BS1) = -2452.222291

E (SMD/B3LYP-D3/BS2//SMD/B3LYP-D3/BS1) = -2453.93232813

C	1.56898700	-1.67137200	-1.68995900
C	2.59155900	-1.47907100	-2.64188600
H	2.84612800	-0.48273100	-2.97385800
C	3.30056800	-2.55173800	-3.17133500
H	4.08153200	-2.37191500	-3.90368000
C	3.00336600	-3.84853900	-2.75243900
H	3.54956100	-4.69735200	-3.15263100
C	1.99649200	-4.05912000	-1.81564100
H	1.77494300	-5.07369300	-1.50516500
C	1.27313700	-2.98176900	-1.28617200
C	0.77257300	-3.94633200	0.88605000
H	1.29585600	-4.86619700	0.60885600

H	-0.02999000	-4.20000400	1.57601200
H	1.46990600	-3.25939900	1.36908900
C	-0.82514400	-4.15416600	-0.97864600
H	-1.20806700	-3.64198300	-1.86337300
H	-1.64437000	-4.34677100	-0.28730600
H	-0.38303100	-5.11097700	-1.27086500
Au	-0.94446700	-1.42136300	0.41109700
N	0.19248000	-3.27382000	-0.31760100
P	0.63318100	-0.24905300	-0.99666200
C	-2.10270900	0.17935500	1.00663700
C	-3.38469300	0.30438500	0.46873400
C	-1.67509500	1.09042000	1.97782500
C	-4.22219200	1.36009100	0.85113700
C	-2.50402600	2.13940600	2.36973500
C	-3.77981800	2.28893200	1.80347700
H	-5.20562900	1.44073300	0.40255400
H	-2.17555600	2.85394600	3.11882500
H	-0.70255200	0.99333100	2.44608600
C	-2.29289300	-2.60067100	1.73302400
H	-3.27814300	-2.32329100	1.35767600
H	-2.04624000	-3.64714500	1.56426300
C	-1.97078400	-2.11307700	3.03492200
H	-2.44525200	-1.18100500	3.34048900
H	-3.74894000	-0.40266300	-0.27151000
C	-1.15488100	-2.74861300	3.98852000
C	-0.54006300	-1.93029500	5.12197800
H	-1.29619400	-1.24081700	5.52156800
H	-0.26578000	-2.60437800	5.94351500
O	-4.50471700	3.34871100	2.24918800
C	-5.81132200	3.55092200	1.70985800
H	-5.77533800	3.72158800	0.62637000
H	-6.46971100	2.69956700	1.92437700
H	-6.20176000	4.44307300	2.20333100
C	0.69554900	-1.13324300	4.67727000
H	0.40064400	-0.45059900	3.87008400
H	1.43617000	-1.82149400	4.24561600
C	1.33048900	-0.33630500	5.81857400
H	1.65281100	-0.99863900	6.63183000
H	2.20847600	0.22242200	5.47375700
H	0.61767200	0.38462400	6.23846100
H	-0.51855800	-3.55010900	3.59887700
H	-2.05668400	-3.43987700	4.56492600
C	-0.15995900	0.67464400	-2.45010400
C	-0.97513200	3.11056000	-3.95753700
H	-1.48458500	3.60329800	-4.79609400
H	-0.51750800	3.89841800	-3.34397400
C	-1.56111600	0.27039300	-4.52416900

H	-2.01961600	-0.51275100	-5.14062600
C	-1.26168900	1.63646400	-1.93293300
H	-1.98925900	1.08052500	-1.34439200
H	-0.83504600	2.40457500	-1.28223500
C	0.10953600	2.15482800	-4.48647700
H	0.84613200	2.71454700	-5.07597000
C	-0.83343900	-0.40481400	-3.34129000
H	-0.08302500	-1.10368100	-3.72694700
H	-1.55111800	-0.98564600	-2.74447200
C	-1.98585900	2.30808300	-3.11915700
H	-2.75346600	2.97860800	-2.71290900
C	-0.53930200	1.06453200	-5.36198400
H	0.23153300	0.38836400	-5.75599800
H	-1.04031500	1.52554000	-6.22315100
C	0.84765300	1.49336900	-3.29882200
H	1.32167700	2.26434700	-2.68291000
H	1.63744800	0.85127800	-3.69603200
C	-2.64664400	1.22450900	-3.99183500
H	-3.18254700	1.68856400	-4.83012100
H	-3.38583900	0.66503900	-3.40149300
C	1.86234800	0.74440700	0.05372300
C	2.37929500	2.08603800	2.64171600
H	3.01870600	2.63433200	3.34556700
H	1.40394500	1.94751400	3.12850000
C	4.18787400	1.71173000	0.34094200
H	5.15504900	1.83753100	-0.16111200
C	1.27755000	2.14429000	0.36921400
H	1.18636600	2.71914800	-0.55827200
H	0.27808600	2.06042400	0.80062100
C	3.00652200	0.71533900	2.31981700
H	3.12176200	0.13293300	3.24206100
C	3.25188900	0.93069200	-0.61291200
H	3.70344000	-0.04294500	-0.82569900
H	3.15609400	1.46993200	-1.56105200
C	2.21253400	2.89600500	1.34065900
H	1.76397800	3.87150700	1.56535400
C	4.37586600	0.91198800	1.64405800
H	4.83321100	-0.06308000	1.42765700
H	5.05484200	1.44856100	2.31952900
C	2.06865600	-0.05659500	1.37003100
H	1.10972000	-0.22369600	1.87144100
H	2.49219300	-1.04374900	1.14020300
C	3.58342800	3.09040500	0.66645400
H	4.25736800	3.64688400	1.33092200
H	3.46916600	3.68079300	-0.25262300
C	-4.29104700	-3.81553100	5.28310200
C	-5.32397400	-4.46318800	5.95530900

C	-5.01485500	-5.58872300	6.72266100
C	-3.69049800	-6.02766800	6.79283200
C	-2.71606900	-5.32415600	6.08949700
N	-3.02543900	-4.24635200	5.35577500
H	-5.79668000	-6.11704900	7.25995400
H	-4.46025800	-2.93218300	4.67044700
H	-6.34067400	-4.09277500	5.87881100
H	-3.41422700	-6.89738800	7.37959600
H	-1.66997100	-5.61596200	6.10435900

9

E (SMD/B3LYP-D3/BS1) = -2204.43334288

G (SMD/B3LYP-D3/BS1) = -2203.588051

E (SMD/B3LYP-D3/BS2//SMD/B3LYP-D3/BS1) = -2205.11099110

C	1.54921300	-1.61898300	-1.66054500
C	2.59860800	-1.44869800	-2.58668600
H	2.88338700	-0.45407900	-2.89959700
C	3.29255700	-2.53349200	-3.11205400
H	4.09453300	-2.36808400	-3.82521800
C	2.94981300	-3.82596800	-2.71438100
H	3.47961800	-4.68606600	-3.11309700
C	1.92120700	-4.01673700	-1.79699300
H	1.66999600	-5.02789000	-1.49748200
C	1.21607400	-2.92614400	-1.26755200
C	0.70789900	-3.86412300	0.90882900
H	1.23860400	-4.78394500	0.64346100
H	-0.09913700	-4.11472800	1.59338000
H	1.39603100	-3.17045800	1.39568600
C	-0.89673000	-4.08208900	-0.93898900
H	-1.28161100	-3.58132500	-1.82965800
H	-1.71301400	-4.24861600	-0.23588200
H	-0.47237400	-5.05166900	-1.21843400
Au	-1.03952800	-1.34041100	0.41215400
N	0.13091900	-3.20245800	-0.29932500
P	0.59175600	-0.18824500	-1.01163700
C	-2.24979800	0.22555500	0.96076500
C	-3.49915700	0.37636100	0.35384200
C	-1.85372700	1.15600200	1.92966600
C	-4.32715500	1.46425400	0.66249200
C	-2.67030900	2.23933100	2.24850900
C	-3.90943500	2.40697800	1.61129200
H	-5.28431700	1.55780400	0.16198500
H	-2.36272300	2.96711300	2.99412300
H	-0.90613900	1.04570600	2.44713200
C	-2.23474000	-2.40714800	1.85582500
H	-3.27960100	-2.14896900	1.67029700
H	-2.07865900	-3.47105700	1.65932500

C	-1.75920000	-1.99139000	3.19621200
H	-2.09654800	-1.01077100	3.53216500
H	-3.84443300	-0.34175400	-0.38517900
C	-0.90417800	-2.66529500	3.98508600
C	-0.31763900	-2.12096500	5.25721400
H	-0.88556800	-1.24063700	5.58828800
H	-0.38363700	-2.86778600	6.06224500
O	-4.62725400	3.50224000	1.98836900
C	-5.90049300	3.71453900	1.38229700
H	-5.81217400	3.84803400	0.29615600
H	-6.58941200	2.88612000	1.59213800
H	-6.29364400	4.63133800	1.82680400
C	1.16307600	-1.72425000	5.07323900
H	1.22653900	-0.96942100	4.27732400
H	1.72999500	-2.59697300	4.71884000
C	1.79852200	-1.17977600	6.35474300
H	1.77289900	-1.92727800	7.15781200
H	2.84620100	-0.89884400	6.19173700
H	1.26457900	-0.28969400	6.71138300
H	-0.54043500	-3.64514400	3.67201300
C	-0.14721700	0.69327600	-2.51759900
C	-0.94939000	3.06932700	-4.12359900
H	-1.45428600	3.53279000	-4.98178200
H	-0.48756900	3.87781000	-3.54046400
C	-1.55199300	0.21418400	-4.57845300
H	-2.01488900	-0.59005200	-5.16417600
C	-1.24639000	1.68212300	-2.04261300
H	-1.97356800	1.15784000	-1.42182200
H	-0.81315000	2.47772700	-1.43062600
C	0.13121500	2.08760200	-4.61407900
H	0.86925600	2.62079800	-5.22652300
C	-0.82961600	-0.41486100	-3.36804800
H	-0.08309700	-1.13377900	-3.72413800
H	-1.55085500	-0.96781300	-2.74933900
C	-1.96741900	2.30760400	-3.25522000
H	-2.73212600	2.99955900	-2.87999300
C	-0.52547100	0.96869500	-5.44687600
H	0.24098900	0.27248500	-5.81415000
H	-1.02332900	1.39882900	-6.32606600
C	0.86321700	1.46610300	-3.40145000
H	1.34712100	2.25682300	-2.81781100
H	1.64657900	0.79889400	-3.77080600
C	-2.63299400	1.19440800	-4.08576900
H	-3.16617100	1.62810500	-4.94228500
H	-3.37528300	0.66223900	-3.47453100
C	1.80367000	0.83523000	0.03175700
C	2.26062100	2.22274000	2.61632600

H	2.88467600	2.78929700	3.32003700
H	1.27992000	2.08192300	3.09181900
C	4.11309200	1.83741800	0.35297700
H	5.08925800	1.97009100	-0.13034900
C	1.19542400	2.23054100	0.32536200
H	1.10590700	2.79702500	-0.60769400
H	0.19110200	2.13330800	0.74636800
C	2.90559500	0.85381500	2.32368400
H	3.01238900	0.28586300	3.25645000
C	3.20368400	1.03215400	-0.60476700
H	3.67109800	0.06107800	-0.79509500
H	3.11987600	1.55505600	-1.56374800
C	2.10385400	3.00972400	1.30045000
H	1.63988700	3.98316200	1.50326000
C	4.28500300	1.05656000	1.66995800
H	4.75591000	0.08352300	1.47375600
H	4.94670500	1.60798200	2.35111000
C	1.98935900	0.06052200	1.36927100
H	1.01969700	-0.10120400	1.85462600
H	2.42101800	-0.92936100	1.16791600
C	3.48433600	3.21243100	0.64771400
H	4.13854400	3.78877400	1.31545700
H	3.37911800	3.78739700	-0.28244900

$TS_{9\text{-}10}$

E (SMD/B3LYP-D3/BS1) = -2204.40272766

G (SMD/B3LYP-D3/BS1) = -2203.562996

E (SMD/B3LYP-D3/BS2//SMD/B3LYP-D3/BS1) = -2205.08037768

C	2.81037400	-0.56739600	-0.93872700
C	3.77043800	-0.47198400	-1.96542500
H	3.46358700	-0.19130600	-2.96507600
C	5.11649800	-0.74184300	-1.73103600
H	5.83281700	-0.66041000	-2.54349200
C	5.53354200	-1.11606700	-0.45237800
H	6.58125300	-1.32475400	-0.25427500
C	4.59715000	-1.22970300	0.57213900
H	4.92031300	-1.52880900	1.56458300
C	3.23727900	-0.97012400	0.34704300
C	2.50645800	-0.15593000	2.51409000
H	3.46956900	-0.29415900	3.03060100
H	1.70179800	-0.26336100	3.24948100
H	2.46838100	0.85512600	2.09927000
C	2.25557100	-2.51176400	1.94691400
H	2.07026200	-3.20079800	1.11810700
H	1.43164900	-2.59580300	2.66231700
H	3.18595400	-2.80934800	2.45557500
Au	-0.24712300	-0.55144800	0.69323800

N	2.29600200	-1.13407400	1.43565200
P	1.03756200	-0.21495100	-1.27324300
C	-2.18647100	0.10740100	1.27961300
C	-3.27373100	-0.31136800	0.51295800
C	-2.22553200	1.35886700	1.91844700
C	-4.36705600	0.53530400	0.30676500
C	-3.30896700	2.20753800	1.71304800
C	-4.38350100	1.80758100	0.89927800
H	-5.19069600	0.19454600	-0.31002200
H	-3.34521000	3.18665600	2.18183500
H	-1.40932900	1.67725600	2.56051600
C	-1.39640700	-1.46361600	2.49915800
H	-0.47317900	-1.26725400	3.05318500
H	-2.24086200	-1.13845700	3.09637300
C	-1.53409400	-2.82617700	1.95131700
H	-0.61745400	-3.34562800	1.67469700
H	-3.27184500	-1.29950100	0.06109900
C	-2.71579500	-3.43645100	1.74429100
C	-2.88196500	-4.81269700	1.17258800
H	-3.47183900	-4.74583500	0.24471400
H	-1.90300000	-5.22789000	0.89714100
O	-5.38902700	2.71261100	0.76255700
C	-6.51066500	2.36296900	-0.04834700
H	-6.21012300	2.16747400	-1.08576400
H	-7.03731000	1.48674800	0.35108300
H	-7.17734700	3.22735600	-0.02242300
C	-3.60374500	-5.77705500	2.13428300
H	-4.57318800	-5.34452600	2.41747900
H	-3.01819400	-5.86304100	3.05942500
C	-3.81473900	-7.16286000	1.51963700
H	-2.85674300	-7.62618100	1.25114300
H	-4.32680500	-7.83426300	2.21909800
H	-4.42199500	-7.10160800	0.60745400
H	-3.63387300	-2.91385100	2.01846100
C	0.45334500	-1.45718200	-2.58691000
C	0.97040900	-3.57667700	-4.59823800
H	0.63802800	-4.36066400	-5.29166600
H	2.06588000	-3.52592700	-4.66405600
C	-1.60026000	-2.66519000	-3.48233000
H	-2.69462200	-2.71275900	-3.41236700
C	0.85447100	-1.11705300	-4.04225800
H	0.41675500	-0.15665900	-4.33507300
H	1.94203300	-1.02715200	-4.13247300
C	0.54432800	-3.93065000	-3.15929700
H	0.99063700	-4.88903200	-2.86499200
C	-1.09649600	-1.57547400	-2.51332000
H	-1.39834900	-1.83200000	-1.49070400

H	-1.56870700	-0.61861300	-2.75678100
C	0.35312100	-2.22250900	-4.99982900
H	0.65963800	-1.96326200	-6.02108300
C	-0.99124200	-4.02186800	-3.08136900
H	-1.30215800	-4.28720700	-2.06112400
H	-1.35602800	-4.81323700	-3.74968400
C	1.05411100	-2.83638500	-2.19767100
H	2.14801700	-2.79892100	-2.23992700
H	0.77467300	-3.08450600	-1.16374400
C	-1.18287600	-2.31152200	-4.92244900
H	-1.55191100	-3.07507900	-5.62023800
H	-1.63121500	-1.35359100	-5.21987300
C	0.90926700	1.60817400	-1.76614600
C	-0.40286600	4.20015800	-1.13428500
H	-0.56131500	5.27430100	-1.29894400
H	-1.06592800	3.89387300	-0.31342400
C	1.63910400	3.58556900	-3.17530700
H	2.30257400	3.88488700	-3.99657100
C	-0.56227400	1.90325400	-2.16057800
H	-0.82176900	1.35077100	-3.07039700
H	-1.24094000	1.56947800	-1.36579600
C	1.06773300	3.93614300	-0.75455500
H	1.32018000	4.48556800	0.16138900
C	1.84648300	2.07617300	-2.90772800
H	2.89109800	1.90973200	-2.62609000
H	1.65572200	1.51084300	-3.82487600
C	-0.75114400	3.41364300	-2.41275100
H	-1.79897000	3.59327500	-2.68494200
C	1.98940400	4.38329300	-1.90487700
H	3.04053700	4.21615300	-1.63254700
H	1.86677600	5.45904500	-2.08909800
C	1.26167900	2.42806000	-0.49127900
H	0.61895200	2.12074700	0.34295700
H	2.30038000	2.22906100	-0.19653600
C	0.17273500	3.85391500	-3.56479300
H	0.03033000	4.92227300	-3.77575900
H	-0.08143100	3.30397000	-4.48144800

10

E (SMD/B3LYP-D3/BS1) = -2204.47782303

G (SMD/B3LYP-D3/BS1) = -2203.636104

E (SMD/B3LYP-D3/BS2//SMD/B3LYP-D3/BS1) = -2205.15151330

C	2.40495500	-0.53894400	-0.75801900
C	3.41223600	-0.60183300	-1.74218000
H	3.15036000	-0.50063300	-2.78745100
C	4.75002300	-0.79808500	-1.41014400
H	5.49945900	-0.84356900	-2.19506300

C	5.11502000	-0.93402400	-0.06977700
H	6.15544000	-1.08358600	0.20552900
C	4.13508000	-0.87624600	0.91736800
H	4.41745700	-0.97983100	1.96097300
C	2.78150500	-0.68384200	0.59841500
C	1.97118100	0.53520100	2.52911400
H	2.90905300	0.51729900	3.10935700
H	1.13194800	0.57537400	3.23064600
H	1.95349400	1.44284900	1.91994600
C	1.74891800	-1.88674400	2.43458100
H	1.57538300	-2.72583700	1.75451000
H	0.90843500	-1.82784000	3.13374200
H	2.66507300	-2.08740800	3.01431100
Au	-0.79493900	-0.42876100	0.53188400
N	1.80975600	-0.63871700	1.66526400
P	0.65549800	-0.27602100	-1.27385700
C	-2.34559600	-0.39126800	2.35473700
C	-3.23332700	-0.20242200	1.26006600
C	-1.83491700	0.78779100	2.97812100
C	-3.60908700	1.07941700	0.82045000
C	-2.18332000	2.05107900	2.53509300
C	-3.07591900	2.20789500	1.45017100
H	-4.29762900	1.16963800	-0.01068500
H	-1.78128200	2.94096300	3.00941100
H	-1.17382600	0.68179600	3.83333400
C	-2.24621000	-1.74672700	3.06429400
H	-1.44674500	-1.68005900	3.81198400
H	-3.18412300	-1.90401600	3.61335400
C	-1.98834600	-2.91214600	2.14287600
H	-0.95631900	-3.02922800	1.80719900
H	-3.70391100	-1.07258400	0.81098000
C	-2.91815800	-3.75730300	1.68390600
C	-2.65017500	-4.88165700	0.72276700
H	-3.26748400	-4.74239100	-0.17839000
H	-1.60283700	-4.84665900	0.39312500
O	-3.33329300	3.48638900	1.08838300
C	-4.25104500	3.72152900	0.01497100
H	-3.89599900	3.27089300	-0.91936300
H	-5.24788700	3.33409100	0.25771100
H	-4.29822200	4.80545600	-0.10013300
C	-2.95507000	-6.27247100	1.30903600
H	-3.99878100	-6.30085700	1.65151100
H	-2.33097300	-6.42909000	2.19931400
C	-2.71086800	-7.39492400	0.29703800
H	-1.66707100	-7.39935400	-0.04269800
H	-2.92585700	-8.37870900	0.73102000
H	-3.34725900	-7.27236200	-0.58881900

H	-3.95005800	-3.64927100	2.02615400
C	0.20978200	-1.61677300	-2.55206300
C	0.92841300	-3.81664000	-4.40888800
H	0.67234300	-4.64763900	-5.07952800
H	2.02341300	-3.72773400	-4.40313700
C	-1.74111300	-2.93838800	-3.52102200
H	-2.83570700	-3.01964800	-3.51969300
C	0.69103500	-1.33801000	-3.99809100
H	0.23588400	-0.41398900	-4.37010700
H	1.77681600	-1.20746800	-4.03248500
C	0.41419900	-4.11105300	-2.98547000
H	0.86772000	-5.03722400	-2.61032300
C	-1.33829700	-1.78293900	-2.57966400
H	-1.70427300	-1.99804900	-1.56841000
H	-1.81709200	-0.85362400	-2.90634000
C	0.29469300	-2.50978100	-4.92472600
H	0.66394400	-2.29175800	-5.93480100
C	-1.11924200	-4.25138600	-3.00947000
H	-1.49326000	-4.47854700	-2.00273600
H	-1.41017900	-5.08605000	-3.66126600
C	0.82216800	-2.95280000	-2.04939600
H	1.91450600	-2.88341400	-2.01710400
H	0.47631400	-3.15631800	-1.02629600
C	-1.23875200	-2.64827000	-4.94765200
H	-1.53453700	-3.46148700	-5.62381400
H	-1.69482900	-1.72418400	-5.32878700
C	0.56096000	1.50860800	-1.91260400
C	-0.76707200	4.14944100	-1.58827100
H	-0.90367700	5.20694700	-1.85123600
H	-1.48565500	3.91622000	-0.79135900
C	1.41378300	3.36241300	-3.41679600
H	2.13750800	3.59059600	-4.20944500
C	-0.87373000	1.77627700	-2.43616500
H	-1.07724000	1.15190800	-3.31358000
H	-1.60853300	1.51069500	-1.66517400
C	0.66953400	3.91182600	-1.08366400
H	0.85972100	4.53108500	-0.19775300
C	1.58611900	1.87842100	-3.01452200
H	2.60512300	1.72636100	-2.64400800
H	1.45635200	1.24571500	-3.89652500
C	-1.02882000	3.26251200	-2.82120800
H	-2.05189100	3.42377300	-3.18501700
C	1.67778500	4.26063600	-2.19410100
H	2.70413300	4.11257500	-1.83142100
H	1.57938200	5.31805500	-2.47409300
C	0.82762800	2.42954800	-0.68662400
H	0.12413100	2.19578700	0.12220800

H	1.83968000	2.25125000	-0.30215200
C	-0.01804000	3.60361200	-3.93370200
H	-0.13395800	4.65173000	-4.24040000
H	-0.20996400	2.98172700	-4.81897400

11

E (SMD/B3LYP-D3/BS1) = -2204.48379037

G (SMD/B3LYP-D3/BS1) = -2203.640773

E (SMD/B3LYP-D3/BS2//SMD/B3LYP-D3/BS1) = -2205.15993799

C	2.61465000	-0.86354700	-1.22659200
C	3.37830000	-0.95995100	-2.40670600
H	2.94589800	-0.65744500	-3.35196600
C	4.68467600	-1.44106300	-2.39364500
H	5.24634600	-1.50449400	-3.32107200
C	5.26216700	-1.83470100	-1.18539200
H	6.28319000	-2.20474900	-1.15739300
C	4.52142700	-1.75579700	-0.00907200
H	4.97359500	-2.06577100	0.92783900
C	3.20054500	-1.28385400	-0.01198500
C	3.05675400	-0.28527300	2.19703700
H	4.07345000	-0.57356100	2.50363900
H	2.42290800	-0.23771800	3.08728200
H	3.09100700	0.70470200	1.73559200
C	2.33949900	-2.59005400	1.84260700
H	1.83810300	-3.25490800	1.13422300
H	1.73134600	-2.51477700	2.74675600
H	3.31264200	-3.02422900	2.11439400
Au	0.03455700	-0.43543500	0.94151800
N	2.46426400	-1.24582900	1.24416400
P	0.89670400	-0.20380900	-1.25896500
C	-3.38409400	1.88130700	2.78177600
C	-3.39683200	1.84463400	4.18058200
C	-4.58873300	2.16122000	2.11851600
C	-4.56343700	2.08651200	4.91079800
C	-5.76072000	2.40447700	2.82732300
C	-5.75751300	2.36883500	4.23090800
H	-4.53028200	2.05498400	5.99377500
H	-6.69171400	2.62668800	2.31402500
H	-4.60721500	2.19416400	1.03123200
C	-2.11449500	1.57092900	2.00748500
H	-2.21157000	1.93268200	0.97871400
H	-1.25325800	2.07883800	2.45724500
C	-1.87831600	0.07235700	2.01027700
H	-2.65851800	-0.51627400	1.52492300
H	-2.47735800	1.62231900	4.71758800
C	-1.03304300	-0.57971600	2.90988400
C	-1.25311400	-2.01304200	3.34304400

H	-1.72869700	-2.57975000	2.53191100
H	-0.29353100	-2.49866400	3.55701800
O	-6.95301100	2.62092600	4.83274900
C	-7.01061400	2.60077400	6.25784200
H	-6.73825300	1.61552900	6.65805700
H	-6.35736100	3.36541000	6.69767600
H	-8.04833300	2.82070000	6.51743500
C	-2.13849400	-2.07827400	4.60286700
H	-3.09911400	-1.59122600	4.38824200
H	-1.66574100	-1.49524200	5.40545700
C	-2.37180400	-3.51551900	5.07449500
H	-1.42302200	-4.01066000	5.31784200
H	-3.00290900	-3.54134900	5.97071100
H	-2.86879000	-4.10962800	4.29702200
H	-0.45728900	0.04082000	3.60043800
C	-0.11133000	-1.25744700	-2.47282100
C	-0.34433100	-3.36370000	-4.54992600
H	-0.92841800	-4.04429300	-5.18365400
H	0.71564300	-3.52215500	-4.79151800
C	-2.47770500	-2.01188600	-3.02962100
H	-3.53508900	-1.85004400	-2.78411700
C	0.11022900	-0.94833100	-3.97374400
H	-0.17532000	0.08731500	-4.18750200
H	1.16508300	-1.06276600	-4.24269800
C	-0.59935100	-3.67942300	-3.06244800
H	-0.31011200	-4.71598500	-2.84785300
C	-1.62314600	-1.07382700	-2.15087100
H	-1.80521400	-1.29906900	-1.09306600
H	-1.92772400	-0.03575800	-2.31815700
C	-0.74098800	-1.90308000	-4.84166600
H	-0.55412900	-1.67176600	-5.89794300
C	-2.09113100	-3.47401300	-2.73794900
H	-2.28382400	-3.71122300	-1.68225100
H	-2.70501700	-4.15506200	-3.34233900
C	0.26079600	-2.73954000	-2.19132400
H	1.32064500	-2.91176700	-2.40878900
H	0.10379800	-2.96582700	-1.12679000
C	-2.23241700	-1.69679400	-4.51766000
H	-2.84970900	-2.35108500	-5.14767100
H	-2.52581700	-0.66100700	-4.73717000
C	1.04542900	1.62749700	-1.70822000
C	0.38649500	4.39840100	-0.85674900
H	0.41919100	5.48813300	-0.98776300
H	-0.18542000	4.19729900	0.05990000
C	1.92072900	3.47715800	-3.20411900
H	2.49232400	3.67427600	-4.11985200
C	-0.38104900	2.21540100	-1.85875000

H	-0.88391200	1.75822000	-2.71822200
H	-0.97729600	1.98404200	-0.96652700
C	1.81680400	3.84012900	-0.72045200
H	2.31336600	4.29560100	0.14580500
C	1.86522600	1.94727300	-2.98364800
H	2.88657900	1.56756500	-2.87713300
H	1.42322300	1.46619800	-3.86100200
C	-0.30726400	3.74239900	-2.06631700
H	-1.32836600	4.13194600	-2.16562300
C	2.61629200	4.14331100	-2.00188300
H	3.64320200	3.76563600	-1.90351800
H	2.68065500	5.22860400	-2.15710600
C	1.74904100	2.31419500	-0.50213300
H	1.20065500	2.10157400	0.42492300
H	2.76162900	1.90811900	-0.38196300
C	0.49285900	4.03826900	-3.34996500
H	0.53169200	5.12104400	-3.52906600
H	-0.00549200	3.58208800	-4.21645000

OTf^-
E (SMD/B3LYP-D3/BS1) = -961.566517519
G (SMD/B3LYP-D3/BS1) = -961.571629
E (SMD/B3LYP-D3/BS2//SMD/B3LYP-D3/BS1) = -961.935672859

S	-7.45053300	-2.97760000	2.90519200
O	-7.79463500	-1.59290300	2.50582600
O	-6.07966500	-3.41549000	2.55173500
O	-7.91617700	-3.38160100	4.25242700
C	-8.51205800	-4.00585400	1.77917800
F	-8.22311200	-3.76905500	0.48447800
F	-9.82069900	-3.73650400	1.96161300
F	-8.33079300	-5.32179800	2.00476700

HOTf
E (SMD/B3LYP-D3/BS1) = -962.002499216
G (SMD/B3LYP-D3/BS1) = -961.997501
G (SMD/B3LYP-D3/BS1) = -962.361152318

S	-7.41256200	-2.87183200	2.79105200
O	-7.84255800	-1.51191300	2.52178000
O	-6.04096200	-3.32250300	2.59848300
O	-7.91884500	-3.23198500	4.28535600
C	-8.51250400	-4.01660800	1.80811000
F	-8.19565400	-3.90025300	0.52115400
F	-9.78498900	-3.68306200	1.99641500
F	-8.30458400	-5.26797000	2.21624400
H	-7.39279400	-3.98222100	4.64580700

1-hexene

E (SMD/B3LYP-D3/BS1) = -235.860722317
G (SMD/B3LYP-D3/BS1) = -235.726505
E (SMD/B3LYP-D3/BS2//SMD/B3LYP-D3/BS1) = -235.958504154

C	-2.57549900	-2.19372200	1.48433900
H	-1.83359900	-2.88759800	1.87435300
H	-2.75463000	-1.29276300	2.07026500
C	-3.23197400	-2.42932300	0.34575400
H	-3.02387200	-3.34796200	-0.20722000
C	-4.28141500	-1.53433500	-0.25446200
H	-3.96438900	-1.22751900	-1.26335300
H	-4.37601100	-0.61630500	0.34119100
C	-5.65489300	-2.22301800	-0.36526000
H	-5.55022100	-3.15237100	-0.94427800
H	-5.98977400	-2.52049000	0.63866800
C	-6.71982300	-1.33430100	-1.01883000
H	-6.37816900	-1.03903500	-2.02134300
H	-6.81869000	-0.40419000	-0.44098700
C	-8.08488100	-2.02065900	-1.12311900
H	-8.46347900	-2.29966600	-0.13127800
H	-8.82889200	-1.36530100	-1.59233300
H	-8.02126200	-2.93761200	-1.72325100

12
E (SMD/B3LYP-D3/BS1) = -2209.66759348
G (SMD/B3LYP-D3/BS1) = -2208.882905
E (SMD/B3LYP-D3/BS2//SMD/B3LYP-D3/BS1) = -2210.36970573

C	1.83415900	-1.66604000	-1.98937900
C	2.89695800	-1.30624700	-2.84249400
H	3.04806200	-0.27083800	-3.11184700
C	3.77121900	-2.25742900	-3.36040100
H	4.58092800	-1.94577500	-4.01366100
C	3.59892200	-3.60349700	-3.03584100
H	4.27120100	-4.35813700	-3.43383000
C	2.55772700	-3.98044200	-2.19286200
H	2.43199600	-5.02843200	-1.94342000
C	1.67979600	-3.02381100	-1.66400400
C	1.17597500	-4.17276700	0.41517100
H	1.72991100	-5.07230500	0.12210400
H	0.36305400	-4.47074200	1.07835900
H	1.84611100	-3.49244700	0.94563100
C	-0.32829800	-4.37047600	-1.49847500
H	-0.76312200	-3.82722900	-2.34053500
H	-1.12385700	-4.68645200	-0.82211900
H	0.18774900	-5.26346500	-1.87202300
N	0.60288700	-3.46756700	-0.76511600
P	0.68250800	-0.41339700	-1.25477500
C	-1.93445400	-0.33157900	0.77587000

C	-3.07046400	0.08950000	0.07535400
C	-1.81203000	0.09301800	2.11301800
C	-4.03603100	0.92938500	0.65395500
C	-2.75859600	0.92750400	2.70480800
C	-3.87806600	1.35862000	1.97587100
H	-4.89342200	1.23430400	0.06384700
H	-2.65055800	1.25293800	3.73622000
H	-0.96559500	-0.22840200	2.71609800
H	-3.23162100	-0.22538800	-0.95162300
O	-4.74524600	2.17776900	2.64245300
C	-5.90026300	2.63718000	1.94683900
H	-5.63063100	3.23200700	1.06383400
H	-6.54475800	1.80351600	1.63757000
H	-6.44438500	3.26867200	2.65285000
C	-0.20105100	0.40096200	-2.72971600
C	-0.87984700	2.64603200	-4.56716500
H	-1.47153000	3.08702600	-5.38068600
H	-0.15142100	3.40320400	-4.24587900
C	-2.09542500	-0.03637700	-4.37099100
H	-2.82712400	-0.78745400	-4.69554600
C	-0.93044200	1.68238600	-2.24984200
H	-1.55398000	1.46747900	-1.37815000
H	-0.19195700	2.43265800	-1.94835300
C	-0.14956100	1.38698500	-5.07064100
H	0.52151300	1.65160200	-5.89773600
C	-1.24278700	-0.63830300	-3.23434000
H	-0.71987700	-1.53513500	-3.59359800
H	-1.89412600	-0.95267700	-2.41224300
C	-1.79472200	2.26282200	-3.38899800
H	-2.31268500	3.15409200	-3.01257600
C	-1.18312300	0.35033000	-5.55007900
H	-0.67183400	-0.54091900	-5.93903200
H	-1.78281100	0.76707300	-6.37042200
C	0.70297800	0.77370500	-3.93433600
H	1.47922300	1.48438700	-3.63384400
H	1.20059100	-0.12007200	-4.32185000
C	-2.82795300	1.21624300	-3.84977900
H	-3.46055100	1.63718800	-4.64275800
H	-3.48836800	0.94750700	-3.01432600
C	1.80147800	0.77344500	-0.26672400
C	2.73300800	1.62435200	2.43825100
H	3.36315700	2.23683200	3.09707400
H	2.11830900	0.97931300	3.08169700
C	3.57590700	2.58702700	-0.22004200
H	4.19080700	3.23296700	-0.85961100
C	0.93423700	1.64348800	0.68093600
H	0.25205400	2.27912600	0.10964500

H	0.32015100	1.00000600	1.31031900
C	3.61539800	0.76343700	1.51474300
H	4.25797500	0.10781300	2.11623500
C	2.67827100	1.71614700	-1.12907800
H	3.31823500	1.14293900	-1.80440800
H	2.04346600	2.36417200	-1.74215500
C	1.82809500	2.52594000	1.57795300
H	1.17795500	3.12728600	2.22616800
C	4.48547700	1.67901300	0.63081800
H	5.12852500	1.07194700	-0.02126600
H	5.14482900	2.29199800	1.25978300
C	2.72486400	-0.11703100	0.61184100
H	2.11281500	-0.78796100	1.23115300
H	3.36144100	-0.74523600	-0.02128100
C	2.69478600	3.44926200	0.70223200
H	3.32372900	4.09084500	1.33407800
H	2.05413300	4.10951100	0.10159200
C	-3.05280100	-3.60244100	0.74209200
C	-3.90438500	-4.39642700	1.50437400
C	-3.56804300	-4.66351600	2.83200800
C	-2.38979300	-4.12324000	3.34869400
C	-1.59183100	-3.33814800	2.52216700
N	-1.91142300	-3.08743700	1.23793200
H	-4.21335300	-5.27736700	3.45309000
H	-3.27896100	-3.36282000	-0.29146700
H	-4.81233300	-4.78975300	1.05956900
H	-2.08889200	-4.29839700	4.37621400
H	-0.67215800	-2.89158400	2.88456400
Pd	-0.68461400	-1.74702500	0.03924800

13

E (SMD/B3LYP-D3/BS1) = -2197.21511299
G (SMD/B3LYP-D3/BS1) = -2196.356913
E (SMD/B3LYP-D3/BS2//SMD/B3LYP-D3/BS1) = -2197.91513521

C	2.24251600	-1.57222900	-1.01375900
C	3.61255200	-1.52009300	-1.34386500
H	4.19100500	-0.63620700	-1.11977300
C	4.25883000	-2.59005300	-1.95489400
H	5.31609100	-2.51624900	-2.19185400
C	3.54093800	-3.74667600	-2.25918700
H	4.02786200	-4.58921500	-2.74153400
C	2.18705800	-3.81769600	-1.94686500
H	1.63582600	-4.71587100	-2.20344900
C	1.53402800	-2.74401600	-1.32359000
C	-0.18459900	-4.08483200	-0.25981000
H	0.14669300	-4.98835700	-0.78214600
H	-1.25733800	-4.17459100	-0.09255200

H	0.33059600	-4.01412600	0.70033300
C	-0.63732800	-2.90353200	-2.35344900
H	-0.39562900	-2.00858300	-2.93040600
H	-1.71196300	-2.93321400	-2.16848900
H	-0.35521000	-3.79224500	-2.93143500
C	1.57108200	1.28870700	-1.51251700
C	1.14056800	0.71722400	-2.89241200
H	0.11657300	0.32340800	-2.82807800
H	1.79358600	-0.11433500	-3.17994300
C	1.21238100	1.81727300	-3.97304100
H	0.91101200	1.38129300	-4.93403500
C	0.62379100	2.46586900	-1.16208900
H	-0.40051200	2.10581600	-1.08802900
H	0.88313900	2.89776700	-0.19195800
C	3.07846000	2.94128200	-2.71156800
H	4.11200700	3.30535200	-2.77117800
C	2.13527500	4.10046700	-2.34270300
H	2.19542500	4.89218200	-3.10158700
H	2.43854300	4.54301600	-1.38394700
C	0.69619300	3.56361700	-2.24500400
H	0.01299100	4.37315700	-1.95797500
C	3.01404800	1.84634500	-1.62061300
H	3.32854800	2.26873800	-0.66045100
H	3.71996700	1.05626300	-1.88452200
C	0.26605900	2.97404200	-3.60114300
H	0.29532400	3.74934800	-4.37844000
H	-0.76923300	2.61005900	-3.54203700
C	2.65876800	2.34204900	-4.06812700
H	3.33812100	1.52431500	-4.34548000
H	2.73003300	3.10586700	-4.85389900
C	2.20186200	0.17721700	1.42338000
C	1.79886600	0.70695800	4.31820800
H	2.16934900	0.86463600	5.33992600
H	0.70371400	0.78182200	4.35428200
C	4.31178500	0.29033800	2.83627800
H	5.40543800	0.21344900	2.78725000
C	1.82503000	1.58612300	1.94902100
H	2.25932600	2.34881000	1.29454000
H	0.74153400	1.72599800	1.93979000
C	2.21922400	-0.68870100	3.81604300
H	1.80765200	-1.46188200	4.47747100
C	3.74943600	0.05891600	1.41339700
H	4.04858100	-0.94054300	1.08495500
H	4.19044900	0.78424000	0.72216000
C	2.37176400	1.78609900	3.37885800
H	2.06903000	2.78100000	3.72913300
C	3.75560700	-0.78706400	3.78617300

H	4.06325300	-1.78553400	3.44631500
H	4.16275000	-0.64592500	4.79633600
C	1.65867500	-0.90856300	2.39521900
H	0.56432900	-0.87949000	2.42364100
H	1.94591400	-1.90406100	2.02991200
C	3.90804900	1.68576500	3.34711300
H	4.32115000	1.85188300	4.35109800
H	4.32204700	2.46350900	2.69076400
N	0.09033900	-2.85283700	-1.04994600
P	1.35391100	-0.13367200	-0.25841400
C	-1.63581000	0.66299900	0.74312100
C	-2.42102600	1.41136500	-0.14234400
C	-1.64751900	1.03889900	2.09565400
C	-3.18891700	2.50290600	0.28673000
C	-2.40396200	2.12304800	2.54132500
C	-3.17922500	2.86661600	1.63915300
H	-3.78170300	3.04976800	-0.43814900
H	-2.40678200	2.40635900	3.59050700
H	-1.07232400	0.48070500	2.82794500
C	-2.39431500	-2.18058100	1.42770300
H	-1.72742200	-2.99417300	1.69827200
H	-2.62740000	-1.46728700	2.21307100
C	-3.09533800	-2.17733700	0.26067300
H	-2.94302400	-2.98915100	-0.44889600
H	-2.45922700	1.15281300	-1.19820200
C	-4.24906200	-1.27061900	-0.05630700
H	-4.09510200	-0.80714600	-1.04025700
H	-4.30840100	-0.46030200	0.67647200
C	-5.57667800	-2.05454400	-0.08226500
H	-5.50302800	-2.87975300	-0.80523900
H	-5.74405500	-2.51383500	0.90194700
O	-3.88200500	3.90871100	2.17241300
C	-4.69646500	4.68575200	1.29861800
H	-4.09920100	5.17543200	0.51812400
H	-5.47854700	4.07615800	0.82688000
H	-5.16434300	5.44883400	1.92453200
C	-6.77081500	-1.16307500	-0.44363300
H	-6.59416600	-0.70559000	-1.42755500
H	-6.83281900	-0.33490000	0.27627500
C	-8.09609100	-1.92955700	-0.46260000
H	-8.31074000	-2.37154100	0.51877600
H	-8.93410700	-1.27202400	-0.72351000
H	-8.07038500	-2.74575800	-1.19604600
Pd	-0.78511100	-1.01992800	0.02409200

$TS_{13}{}^{i}$

E (SMD/B3LYP-D3/BS1) = -2197.18607621

G (SMD/B3LYP-D3/BS1) = -2196.325795
E (SMD/B3LYP-D3/BS2//SMD/B3LYP-D3/BS1) = -2197.88377373

C	1.49182900	-1.91052500	-1.29828100
C	2.78098600	-2.23557300	-1.76860100
H	3.62792700	-1.63481500	-1.47177600
C	3.00640000	-3.32125700	-2.60864100
H	4.01429600	-3.54155500	-2.94809600
C	1.93098900	-4.11328600	-3.01081800
H	2.08358200	-4.95922700	-3.67483100
C	0.64914000	-3.81039100	-2.56325900
H	-0.18000100	-4.41994800	-2.90433000
C	0.42042500	-2.72415900	-1.70442900
C	-1.62118300	-3.63664700	-0.73864700
H	-1.56433300	-4.49058700	-1.42105200
H	-2.67396100	-3.42544800	-0.55993000
H	-1.13189800	-3.89493900	0.20301500
C	-1.71231200	-1.97852000	-2.53112000
H	-1.19182900	-1.12768800	-2.97357900
H	-2.72301100	-1.67910400	-2.25444500
H	-1.77119500	-2.78809700	-3.26925000
C	1.76151800	1.04912000	-1.30669000
C	1.06077000	0.90730200	-2.68730800
H	-0.02625300	0.83870300	-2.54529400
H	1.38534100	-0.01461300	-3.18391600
C	1.39206400	2.11790300	-3.58604500
H	0.89271100	1.98171800	-4.55404100
C	1.26483500	2.36800600	-0.65556300
H	0.18794900	2.31663700	-0.49817700
H	1.72943400	2.51774800	0.32067600
C	3.61290500	2.37136600	-2.42779600
H	4.70020100	2.41937000	-2.56994600
C	3.11610600	3.66594600	-1.75679200
H	3.36816000	4.53385200	-2.38129100
H	3.61635600	3.80469400	-0.78836200
C	1.59220800	3.57799000	-1.55460600
H	1.22689000	4.48540800	-1.05567400
C	3.29034600	1.15271200	-1.53063400
H	3.81122800	1.25015400	-0.57213100
H	3.67053300	0.25372400	-2.02257600
C	0.89247400	3.41184200	-2.91583100
H	1.10384100	4.27612000	-3.56003200
H	-0.19647400	3.36794100	-2.77412100
C	2.91761400	2.19852500	-3.79283300
H	3.27907800	1.28611000	-4.28691700
H	3.16336900	3.04383500	-4.44973400
C	2.16936000	-0.66928300	1.35361100
C	2.19025800	-0.39120800	4.31865300

H	2.65066100	-0.52595800	5.30659700
H	1.22622200	0.11299800	4.47523900
C	4.21764100	-1.59259900	2.54678400
H	5.17773300	-2.09729600	2.37908300
C	2.43582400	0.69102500	2.04913700
H	3.10127200	1.29993300	1.42909600
H	1.50606800	1.24977600	2.17200900
C	1.96626400	-1.76051900	3.64766100
H	1.31309900	-2.38046100	4.27544200
C	3.53123500	-1.38730700	1.17602500
H	3.38532100	-2.36681900	0.71075100
H	4.18741200	-0.80152500	0.52208200
C	3.10039800	0.47395700	3.42585600
H	3.25559600	1.45442100	3.89413700
C	3.31969600	-2.46662800	3.44285000
H	3.16475500	-3.44980200	2.97745000
H	3.80658800	-2.63764000	4.41240700
C	1.28146900	-1.54616100	2.28192900
H	0.30836500	-1.06241800	2.43303100
H	1.09076400	-2.51653200	1.80312400
C	4.45446000	-0.23139000	3.22651700
H	4.95470400	-0.37492500	4.19378200
H	5.11455700	0.39102900	2.60681500
N	-0.97186000	-2.42137100	-1.30844000
P	1.15532300	-0.41961700	-0.25478400
C	-1.63978800	1.18199900	1.00527600
C	-2.27542300	2.02323100	0.07374400
C	-0.96045000	1.78060300	2.08157800
C	-2.14627200	3.41285000	0.13911200
C	-0.83326700	3.16061100	2.16632500
C	-1.40068200	3.98860200	1.17994100
H	-2.62579600	4.02846900	-0.61312800
H	-0.28530300	3.62346000	2.98204300
H	-0.52597900	1.15833300	2.85756000
C	-2.96088100	-0.21921700	1.78050500
H	-2.43958700	-0.45319000	2.70499700
H	-3.68153000	0.58632200	1.86911000
C	-3.19290600	-1.24641200	0.84028800
H	-2.90184900	-2.24870700	1.15583500
H	-2.87818900	1.59550900	-0.72338300
C	-4.37698400	-1.15833500	-0.09584600
H	-4.24612400	-1.84317600	-0.94116000
H	-4.44611900	-0.14342500	-0.51040200
C	-5.70183600	-1.50680500	0.61198200
H	-5.62628000	-2.51657100	1.04111100
H	-5.84889700	-0.81875900	1.45663200
O	-1.18422800	5.32062600	1.33087300

C	-1.69656000	6.20882300	0.33628000
H	-1.28400900	5.97923400	-0.65441800
H	-2.79249100	6.17448000	0.29368300
H	-1.37833500	7.20842200	0.63865200
C	-6.91280000	-1.43837200	-0.32555800
H	-6.75457100	-2.12405700	-1.17039100
H	-6.97943600	-0.42842700	-0.75502700
C	-8.22821300	-1.78531100	0.37753700
H	-8.42552600	-1.09588500	1.20846600
H	-9.07830500	-1.72843100	-0.31306800
H	-8.19943700	-2.80222900	0.78955400
Pd	-1.20191800	-0.66982600	0.10786500

TS_{13}ii

E (SMD/B3LYP-D3/BS1) = -2197.18948105
G (SMD/B3LYP-D3/BS1) = -2196.329976
E (SMD/B3LYP-D3/BS2//SMD/B3LYP-D3/BS1) = -2197.88645790

C	1.41802100	-1.94461200	-1.28912300
C	2.66269600	-2.31581500	-1.83630500
H	3.54154700	-1.72560200	-1.61612100
C	2.79851200	-3.43765400	-2.64798800
H	3.77111700	-3.69651800	-3.05630400
C	1.67853800	-4.22151100	-2.92945600
H	1.76214200	-5.09530400	-3.56925100
C	0.44697100	-3.89236600	-2.37108800
H	-0.40832300	-4.52481100	-2.58223000
C	0.31024200	-2.77011800	-1.53959300
C	-1.36986200	-3.67286900	-0.05451600
H	-1.39754100	-4.60447200	-0.63056500
H	-2.35901400	-3.50902200	0.37241900
H	-0.63633600	-3.76731500	0.74919800
C	-2.03692600	-2.31644000	-1.97593200
H	-1.76022300	-1.45782100	-2.59160600
H	-3.00272900	-2.12959200	-1.50664300
H	-2.12620900	-3.20414000	-2.61304200
C	1.77119500	1.02298200	-1.28047400
C	0.68187200	1.21058500	-2.37484100
H	-0.30509600	1.30727300	-1.91043500
H	0.65493000	0.32619700	-3.02568300
C	0.97746100	2.47111200	-3.21901000
H	0.18897800	2.57722000	-3.97556300
C	1.79473500	2.28505500	-0.37973600
H	0.83825300	2.39807000	0.13614000
H	2.56990600	2.18395800	0.38816100
C	3.43573700	2.16249900	-2.82878100
H	4.41598500	2.04013900	-3.30736000
C	3.45646900	3.39626900	-1.90635000

H	3.69023500	4.29783800	-2.48875500
H	4.24314200	3.28550800	-1.14707400
C	2.08237900	3.54358500	-1.22682800
H	2.08265100	4.41731500	-0.56213900
C	3.13713000	0.89025300	-2.00288600
H	3.94631400	0.72255300	-1.28774200
H	3.11511100	0.03730400	-2.68695300
C	0.99148700	3.71022300	-2.30248200
H	1.18635400	4.61346800	-2.89688500
H	0.01302500	3.83624400	-1.82576500
C	2.34809500	2.33149500	-3.90606900
H	2.34850200	1.46350800	-4.57985100
H	2.55460800	3.22000800	-4.51810900
C	2.33365700	-0.79387500	1.28499500
C	2.43776300	-1.73647500	4.12084800
H	2.98918500	-1.97125700	5.04125600
H	1.37715200	-1.95241100	4.31231400
C	4.62784400	-0.82420900	2.36662400
H	5.68947300	-0.61089500	2.18761000
C	1.81296800	0.05272700	2.47499900
H	1.86441500	1.12195600	2.24455600
H	0.75949900	-0.19122000	2.64663500
C	2.95379800	-2.60646200	2.95939900
H	2.81756300	-3.66869600	3.19987300
C	3.83859200	-0.49730100	1.07848900
H	4.23821000	-1.09227000	0.25079200
H	3.98360800	0.55818000	0.82759000
C	2.61566600	-0.25036800	3.75694400
H	2.23056300	0.38081400	4.56845900
C	4.44835900	-2.31295700	2.72145400
H	4.83054300	-2.94284700	1.90635900
H	5.02775100	-2.55800400	3.62178200
C	2.16442400	-2.29023600	1.67035900
H	1.09902000	-2.51741800	1.81734100
H	2.52557300	-2.93557800	0.86305700
C	4.10750500	0.05221000	3.52184000
H	4.68170800	-0.14836700	4.43640000
H	4.24287400	1.11503000	3.27816200
N	-1.00463500	-2.50667700	-0.91198800
P	1.20678800	-0.45521000	-0.22089400
C	-1.51423800	1.33179500	1.06925600
C	-2.00016700	2.15391800	0.03255800
C	-0.92093900	1.97222200	2.17834800
C	-1.83691700	3.53775300	0.04768600
C	-0.75812000	3.34963000	2.21618900
C	-1.19397200	4.14441400	1.13953900
H	-2.21515400	4.12966800	-0.77740100

H	-0.29181900	3.83724700	3.06740800
H	-0.59487100	1.38752600	3.03106600
C	-3.07871500	0.17743600	1.84075100
H	-2.63736100	0.06084400	2.82934200
C	-3.08856200	-0.98767000	1.03011000
H	-2.88642000	-1.93243500	1.53422800
H	-2.53605700	1.70952100	-0.80247300
O	-0.96706700	5.47455500	1.25871300
C	-1.36285700	6.33510200	0.18735800
H	-0.85313500	6.06782400	-0.74644100
H	-2.44887900	6.31016800	0.03482600
H	-1.06309300	7.34028300	0.48961400
C	-4.20466100	1.18461700	1.78962800
H	-3.87165500	2.14861800	2.18855900
H	-4.51964900	1.34101500	0.75188000
C	-5.39997100	0.67054800	2.61757400
H	-5.73996800	-0.29205700	2.21170100
H	-5.07526300	0.47948300	3.65047600
C	-6.56721900	1.66612600	2.62636000
H	-6.88341200	1.86001300	1.59168400
H	-6.21895800	2.62739700	3.02938200
C	-7.76106300	1.16623600	3.44458300
H	-8.58290500	1.89232800	3.43552600
H	-8.14625800	0.21974100	3.04402100
H	-7.47847800	0.99372800	4.49097900
H	-3.82227900	-1.03407800	0.22417500
Pd	-1.10998200	-0.60668800	0.31219300

TS_{13}^{iii}

E (SMD/B3LYP-D3/BS1) = -3158.76098586

G (SMD/B3LYP-D3/BS1) = -3157.891029

E (SMD/B3LYP-D3/BS2//SMD/B3LYP-D3/BS1) = -3159.81399988

C	1.70067300	-1.54457300	-1.50394500
C	2.75235100	-1.40730400	-2.43263600
H	2.97166300	-0.43735400	-2.85497000
C	3.52902900	-2.49150600	-2.82982300
H	4.32815100	-2.34882800	-3.55172300
C	3.27252200	-3.75391200	-2.29344700
H	3.86966200	-4.61239100	-2.58801800
C	2.24090700	-3.91267500	-1.37344500
H	2.05196800	-4.89789700	-0.96154800
C	1.44805900	-2.82339300	-0.97985100
C	0.95804800	-3.46091700	1.29178200
H	1.54584800	-4.38313900	1.19320400
H	0.16295700	-3.63328200	2.01712900
H	1.60493800	-2.65939800	1.65486900
C	-0.54526500	-4.11535600	-0.52303700

H	-0.86528800	-3.84169100	-1.52772200
H	-1.41483500	-4.19093400	0.12776100
H	-0.05200700	-5.09504600	-0.55757700
N	0.36226600	-3.05222100	-0.01068800
P	0.63328700	-0.12165200	-0.98721800
C	-2.02374500	0.44721200	0.99115300
C	-3.21963900	0.81115600	0.35172800
C	-1.64269400	1.23733800	2.09380500
C	-3.98021600	1.92248000	0.74382300
C	-2.38428000	2.34811900	2.50320200
C	-3.55680300	2.70573300	1.82433600
H	-4.89269300	2.15641400	0.20548100
H	-2.06518900	2.95184700	3.34937500
H	-0.74001000	0.99887100	2.65088000
C	-2.22293500	-2.25445700	1.85053800
H	-1.78947800	-3.24639300	1.99895500
H	-2.13655600	-1.64501400	2.75445800
C	-3.56992700	-2.29041500	1.33446800
H	-4.14074500	-1.36703800	1.44287300
H	-3.58955900	0.21737500	-0.48143600
C	-4.18493400	-3.29444500	0.61786200
C	-5.66453600	-3.27694200	0.28520300
H	-6.00695000	-2.23652600	0.18920800
H	-5.83330500	-3.75218400	-0.68974200
O	-4.21071200	3.81480400	2.29360200
C	-5.41061100	4.20741700	1.63759800
H	-5.23493200	4.45339900	0.58137900
H	-6.18236300	3.42808200	1.70120300
H	-5.76098500	5.10109200	2.15962100
C	-6.52604900	-3.99910800	1.33670300
H	-6.38482400	-3.50860900	2.30983500
H	-6.16091500	-5.02942300	1.45288900
C	-8.01241300	-4.01655700	0.96986800
H	-8.17571600	-4.52696600	0.01179800
H	-8.60831600	-4.53486600	1.73109500
H	-8.40687400	-2.99661300	0.87321400
H	-3.70079900	-4.27355000	0.58038300
H	-3.59980000	-2.71271800	-0.69581200
C	-0.03818000	0.61158800	-2.61760700
C	-0.89661100	2.81558400	-4.44099400
H	-1.38479600	3.18354800	-5.35407500
H	-0.50839500	3.69186900	-3.90313400
C	-1.31747200	-0.09607000	-4.69905200
H	-1.71241900	-0.97004700	-5.23151700
C	-1.21552500	1.56347100	-2.27734900
H	-1.94101200	1.03459400	-1.66140300
H	-0.86723100	2.42227600	-1.69614300

C	0.25860600	1.86439100	-4.80470400
H	0.99518300	2.39327400	-5.42382300
C	-0.61509500	-0.58818000	-3.41719900
H	0.18723700	-1.28236100	-3.69210800
H	-1.32467200	-1.14467900	-2.79691000
C	-1.90984000	2.06154000	-3.56136400
H	-2.72854500	2.73431200	-3.27308100
C	-0.29659400	0.65128600	-5.57913000
H	0.52399700	-0.02394400	-5.85924200
H	-0.77272600	0.98836600	-6.51028800
C	0.96255800	1.38369500	-3.51257400
H	1.36192500	2.25029500	-2.97452400
H	1.80843600	0.75085100	-3.79333200
C	-2.47319400	0.85277600	-4.32931000
H	-2.99388300	1.18567500	-5.23813600
H	-3.20636600	0.32370500	-3.70866900
C	1.82208000	1.05814300	-0.07757700
C	2.22110800	2.67270600	2.38977900
H	2.81465000	3.33371300	3.03610700
H	1.25972700	2.50172700	2.89260000
C	4.06043600	2.24701900	0.12345600
H	5.01778700	2.41262300	-0.38781700
C	1.12153200	2.42542000	0.13198300
H	0.96958400	2.91403000	-0.83631400
H	0.13650000	2.28417300	0.58682100
C	2.95758300	1.33375300	2.18615700
H	3.12104600	0.84804500	3.15712700
C	3.19628300	1.30777700	-0.75118400
H	3.73040800	0.36074700	-0.87612700
H	3.06835400	1.74884700	-1.74492300
C	1.98646800	3.34273000	1.02211600
H	1.45718400	4.29418200	1.16196700
C	4.30956300	1.58667400	1.49297100
H	4.84864900	0.63819600	1.36173300
H	4.93915700	2.23620100	2.11653100
C	2.09357800	0.40543300	1.30782300
H	1.14700800	0.19169600	1.81598000
H	2.60609300	-0.55552200	1.16540100
C	3.33796200	3.59241100	0.32625600
H	3.95968400	4.26503900	0.93304100
H	3.17605000	4.08428400	-0.64301000
S	-3.39201600	-3.47489200	-2.82384100
O	-3.83296700	-4.75485100	-2.26558400
O	-3.25153100	-2.37794100	-1.72174000
O	-2.25165900	-3.43509200	-3.73754000
C	-4.83656100	-2.79637500	-3.78516100
F	-5.86301300	-2.56053100	-2.96195200

F	-5.20446900	-3.69039500	-4.70637100
F	-4.49028000	-1.65715700	-4.39186700
Pd	-0.94152200	-1.17971000	0.44491000

pyridine
E (SMD/B3LYP-D3/BS1) = -248.293591142
G (SMD/B3LYP-D3/BS1) = -248.231852
E (SMD/B3LYP-D3/BS2//SMD/B3LYP-D3/BS1) = -248.393214813

C	-0.48187500	-3.91893800	3.05407600
C	-1.12092600	-4.67041800	0.98689300
C	0.51371600	-4.89212600	3.16074600
H	-0.63594700	-3.20231700	3.85934900
C	-0.15593900	-5.67970700	0.99486400
H	-1.78773600	-4.55692300	0.13351500
C	0.67976900	-5.79242000	2.10705600
H	1.13930800	-4.93904500	4.04710700
H	-0.06580500	-6.35627600	0.15026200
H	1.44335300	-6.56437500	2.15178000
N	-1.29290400	-3.79766900	1.99168100

pyridine.H+
E (SMD/B3LYP-D3/BS1) = -248.750401424
G (SMD/B3LYP-D3/BS1) = -248.674666
E (SMD/B3LYP-D3/BS2//SMD/B3LYP-D3/BS1) = -248.846882681

C	-0.46656200	-3.90810400	3.09302000
C	-1.12950400	-4.68795100	0.94838900
C	0.51236500	-4.88425800	3.17030400
H	-0.65698100	-3.17567100	3.86703300
C	-0.16320900	-5.67883100	0.98502200
H	-1.81404200	-4.53639100	0.12347000
C	0.66569700	-5.77787900	2.10629000
H	1.14115700	-4.93836600	4.05108600
H	-0.06661600	-6.35853300	0.14668000
H	1.42835200	-6.54889100	2.15035600
N	-1.24772200	-3.84363800	1.99448500
H	-1.96336800	-3.11891600	1.95176200

TS$_{2-14}$
E (SMD/B3LYP-D3/BS1) = -3166.46097054
G (SMD/B3LYP-D3/BS1) = -3165.582703
E (SMD/B3LYP-D3/BS2//SMD/B3LYP-D3/BS1) = -3167.49027732

C	2.19806300	-1.54578500	-1.01034500
C	3.57744900	-1.58948400	-1.29777600
H	4.20224000	-0.72989200	-1.10201400
C	4.17045300	-2.72805300	-1.83322900
H	5.23612100	-2.73346200	-2.04053500

C	3.38816700	-3.85133300	-2.09900000
H	3.83370000	-4.74791500	-2.51925100
C	2.02071600	-3.82027900	-1.84204600
H	1.42364500	-4.68964500	-2.09167500
C	1.41812500	-2.67680000	-1.30049700
C	-0.54789500	-3.86720700	-0.43518400
H	-0.19256400	-4.78553100	-0.90821700
H	-1.63390200	-3.88359100	-0.47448800
H	-0.20321800	-3.83210600	0.59971600
C	-0.63696100	-2.61151400	-2.53674100
H	-0.22488800	-1.75517800	-3.07179700
H	-1.71917000	-2.51510500	-2.47148300
H	-0.39423100	-3.53171900	-3.07889200
C	1.71413100	1.37702800	-1.52267000
C	1.31519900	0.83289700	-2.92190700
H	0.27301600	0.48587000	-2.89955100
H	1.94337800	-0.02241200	-3.19380000
C	1.47042100	1.94426800	-3.98255300
H	1.19398200	1.53032800	-4.96014800
C	0.78642400	2.57299400	-1.18767900
H	-0.24923600	2.23794400	-1.16780000
H	1.01351600	2.97998200	-0.19901900
C	3.32034800	2.98896300	-2.63076900
H	4.36594600	3.32054300	-2.64367300
C	2.39945400	4.17184200	-2.27965900
H	2.51491900	4.97232400	-3.02221600
H	2.67774800	4.58934100	-1.30250400
C	0.94007400	3.68275900	-2.24985600
H	0.27286900	4.51011000	-1.97765800
C	3.17987400	1.88370300	-1.55818000
H	3.46258700	2.28337000	-0.57906200
H	3.86882800	1.07125200	-1.80084800
C	0.54841000	3.12699700	-3.63173900
H	0.63691000	3.91199400	-4.39412400
H	-0.50026500	2.79807600	-3.62272800
C	2.93624000	2.42025700	-4.01080500
H	3.59889600	1.58397300	-4.27190400
H	3.06390800	3.19100300	-4.78206000
C	2.18822400	0.19628300	1.43149600
C	1.69953300	0.67935200	4.31364700
H	2.03844500	0.80221600	5.35057700
H	0.60742200	0.79286400	4.31239000
C	4.24859100	0.20511000	2.91320600
H	5.33955600	0.09037600	2.89866100
C	1.83893000	1.60731400	1.96814200
H	2.32141900	2.36606500	1.34355800
H	0.76105900	1.78248100	1.92651000

C	2.08999100	-0.71997700	3.79747700
H	1.62806000	-1.49187500	4.42556600
C	3.73066500	0.02236500	1.46670100
H	4.00450300	-0.98049300	1.12709000
H	4.21848400	0.74579600	0.80612100
C	2.34305200	1.75726100	3.41982400
H	2.06191100	2.75453200	3.78012600
C	3.62201100	-0.87171500	3.81861800
H	3.90725800	-1.87293300	3.46817200
H	3.99650400	-0.76530200	4.84515200
C	1.57292700	-0.89362200	2.35344700
H	0.48024900	-0.82651900	2.35184300
H	1.84049100	-1.88929600	1.97590100
C	3.87511000	1.60364800	3.43811800
H	4.25733300	1.73474500	4.45902100
H	4.33894300	2.37909400	2.81347500
Au	-0.86055900	-0.78824400	-0.06488700
N	-0.05304400	-2.65456500	-1.15527400
P	1.42700300	-0.03927200	-0.29440900
C	-1.65993900	0.91498400	0.76787900
C	-2.30934900	1.83083700	-0.05747800
C	-1.65216400	1.12211000	2.15184800
C	-2.91628800	2.97466000	0.47842400
C	-2.25011400	2.25716400	2.69352400
C	-2.88010000	3.19682700	1.86122600
H	-3.40563900	3.67217000	-0.19156300
H	-2.24004800	2.42977400	3.76573300
H	-1.18740700	0.40722700	2.82149300
C	-2.78279800	-1.67646400	0.63238800
H	-2.38623200	-2.60724900	1.02960400
H	-3.18963100	-1.02788500	1.40386200
C	-3.57708800	-1.79973600	-0.53755900
H	-3.27184700	-2.52688900	-1.27993500
H	-2.35068600	1.68434400	-1.13239300
C	-4.50470400	-0.73484400	-1.00608000
H	-3.84392100	0.04752500	-1.41387200
H	-5.01194500	-0.27586000	-0.14963700
C	-5.49675600	-1.15733600	-2.09524900
H	-4.95154400	-1.65826800	-2.90820600
H	-6.18913200	-1.89646400	-1.67929200
O	-3.42450900	4.27282200	2.48953700
C	-4.07047400	5.26572600	1.69292200
H	-3.37427000	5.71906000	0.97556600
H	-4.93166200	4.85028800	1.15407700
H	-4.41675000	6.02932500	2.39228400
C	-6.28027800	0.03340700	-2.65837700
H	-5.57739700	0.76654100	-3.08063200

H	-6.80157000	0.54354000	-1.83601800
C	-7.29371200	-0.38397600	-3.72719900
H	-8.02516000	-1.09322400	-3.31976700
H	-7.84523400	0.48096200	-4.11480000
H	-6.79568500	-0.87167400	-4.57508800
S	-4.85335400	-4.63141800	-0.37184000
O	-5.02967600	-3.20555900	0.10542600
O	-3.79685400	-4.78056800	-1.39529200
O	-6.12415100	-5.33163900	-0.60345000
C	-4.13497800	-5.46040900	1.13098500
F	-2.93736600	-4.92171100	1.44142200
F	-3.95545400	-6.76621300	0.89627600
F	-4.94558000	-5.31626700	2.18563900

14

E (SMD/B3LYP-D3/BS1) = -3166.47226688

G (SMD/B3LYP-D3/BS1) = -3165.594058

E (SMD/B3LYP-D3/BS2//SMD/B3LYP-D3/BS1) = -3167.49372032

C	2.20385900	-1.55433500	-0.97705900
C	3.57280300	-1.56589500	-1.31095200
H	4.17311600	-0.68061400	-1.15231500
C	4.18054700	-2.69436800	-1.85173500
H	5.23855200	-2.67431900	-2.09501200
C	3.41991800	-3.84079100	-2.08030200
H	3.87578700	-4.73056100	-2.50428000
C	2.05965200	-3.84097700	-1.78415900
H	1.47849500	-4.72787200	-2.00905700
C	1.44294600	-2.70767900	-1.23562300
C	-0.45841000	-3.93995300	-0.29691300
H	-0.13426800	-4.85906000	-0.79208000
H	-1.54344100	-3.95138600	-0.24908300
H	-0.04465900	-3.90172200	0.71268700
C	-0.65213400	-2.71270500	-2.40705100
H	-0.30362200	-1.83412600	-2.94947900
H	-1.73466000	-2.67116800	-2.31042900
H	-0.37846900	-3.61603800	-2.96347200
C	1.56185600	1.31161300	-1.56467300
C	1.21169900	0.68606200	-2.94288500
H	0.21421100	0.22787600	-2.89696300
H	1.92640300	-0.10384800	-3.19788300
C	1.23247500	1.77185600	-4.04063100
H	0.99173100	1.29852500	-5.00082100
C	0.50520300	2.40498400	-1.26346300
H	-0.48470800	1.94633400	-1.23737200
H	0.67609100	2.85955800	-0.28364000
C	2.97333700	3.04862900	-2.74816800
H	3.97570800	3.49314000	-2.78616100

C	1.93227200	4.13448000	-2.41537900
H	1.95398200	4.92346900	-3.17876100
H	2.17365300	4.60506100	-1.45250800
C	0.53216200	3.49537400	-2.35511700
H	-0.21595300	4.25685600	-2.10087900
C	2.96283100	1.96715100	-1.64356600
H	3.21993600	2.42708600	-0.68453700
H	3.72803000	1.21724200	-1.86814100
C	0.19151900	2.85981200	-3.71599300
H	0.18982600	3.62576300	-4.50267100
H	-0.81614200	2.42175200	-3.68530700
C	2.63707900	2.40308000	-4.10630700
H	3.38357100	1.63609500	-4.35403500
H	2.67064500	3.16000700	-4.90097000
C	2.27717700	0.29522200	1.38315300
C	1.90566100	0.80119000	4.28411000
H	2.28872900	0.98689100	5.29625400
H	0.80972500	0.79868800	4.34464400
C	4.40574000	0.56185200	2.74200300
H	5.50047100	0.56390000	2.66706600
C	1.80537500	1.67341300	1.91686700
H	2.15586100	2.46855500	1.25060500
H	0.71375600	1.72146800	1.94343100
C	2.41300500	-0.56202000	3.77309100
H	2.07352800	-1.36144700	4.44386900
C	3.82808000	0.28500700	1.33356800
H	4.18590800	-0.69408300	1.00245900
H	4.20159000	1.03396400	0.62899400
C	2.37602000	1.91662400	3.33050400
H	2.01083400	2.88761100	3.68783800
C	3.95118500	-0.54989500	3.70630100
H	4.32189200	-1.52452600	3.36043000
H	4.37026600	-0.37690900	4.70645300
C	1.83499300	-0.82646300	2.36598300
H	0.74330800	-0.87448000	2.42955000
H	2.18364100	-1.79936600	1.99447300
C	3.91415100	1.92700000	3.25818500
H	4.33933400	2.12762300	4.25063300
H	4.25411900	2.72929900	2.58897700
Au	-0.86495100	-0.89265200	0.11247300
N	-0.02572600	-2.72518200	-1.04668900
P	1.40419400	-0.06096600	-0.26528500
C	-1.66404700	0.71156200	1.11990800
C	-2.36128400	1.70804900	0.43830400
C	-1.58462800	0.77552500	2.51774200
C	-2.91396300	2.79854200	1.12353400
C	-2.13071400	1.85438400	3.20906300

C	-2.79146100	2.88109700	2.51638500
H	-3.43829300	3.56180300	0.55995700
H	-2.05600300	1.91482600	4.29099200
H	-1.09280300	-0.00856700	3.08300700
C	-2.74772100	-1.76759300	0.63581700
H	-2.49325300	-2.68376700	1.17685700
H	-3.26967700	-1.10064000	1.32181200
C	-3.64278000	-2.05877600	-0.54212200
H	-3.26770400	-2.87419300	-1.15993900
H	-2.49436000	1.65857400	-0.63669400
C	-4.01494700	-0.86475100	-1.40753800
H	-3.07586300	-0.49152800	-1.83808200
H	-4.40259200	-0.06634100	-0.76326900
C	-5.00470300	-1.17558500	-2.53340100
H	-4.63823500	-2.02779800	-3.12282200
H	-5.96552500	-1.48571800	-2.10284200
O	-3.28314600	3.89405900	3.28188500
C	-3.99396000	4.94638900	2.63167000
H	-3.35632400	5.47917700	1.91425300
H	-4.88650600	4.56954000	2.11563400
H	-4.29861000	5.63396200	3.42336500
C	-5.23057800	0.02763900	-3.45655800
H	-4.26920000	0.33184900	-3.89604200
H	-5.58050300	0.88082000	-2.85810200
C	-6.23657000	-0.26351500	-4.57317300
H	-7.21498800	-0.53857000	-4.15931100
H	-6.38026900	0.60973000	-5.22079400
H	-5.89726500	-1.09584100	-5.20310100
S	-5.51497300	-4.00756400	-0.30731900
O	-4.94724400	-2.56658800	0.05936900
O	-4.97765300	-4.50593600	-1.57013900
O	-6.94430000	-3.99468700	-0.03316500
C	-4.72091100	-5.02997700	1.03798000
F	-3.39656000	-5.07590300	0.83788800
F	-5.21613700	-6.26477000	0.98964200
F	-4.96821800	-4.48768200	2.22774300

TS_{14}

E (SMD/B3LYP-D3/BS1) = -3166.44950086

G (SMD/B3LYP-D3/BS1) = -3165.571397

E (SMD/B3LYP-D3/BS2//SMD/B3LYP-D3/BS1) = -3167.46283574

C	2.80579500	-0.66677600	-0.64134500
C	3.91840400	-0.48554600	-1.48652500
H	3.77194300	-0.11423500	-2.49310000
C	5.21047900	-0.78402000	-1.06306900
H	6.04861100	-0.63462900	-1.73750200
C	5.41620500	-1.27687900	0.22696800

H	6.41894900	-1.51180900	0.57291700
C	4.32774000	-1.47490600	1.07238700
H	4.49199200	-1.86677900	2.07131200
C	3.02074800	-1.18218300	0.65495400
C	2.00615400	-0.58675800	2.77480800
H	2.88886600	-0.81988200	3.38826000
H	1.11385500	-0.74772200	3.38821400
H	2.04539700	0.46518800	2.47925400
C	1.80025800	-2.85930800	1.91739800
H	1.67009300	-3.44137500	1.00278800
H	0.91697000	-2.99404600	2.54331800
H	2.67929600	-3.23257500	2.46339600
Au	-0.40990500	-0.79055500	0.54316600
N	1.91592000	-1.42922500	1.56927300
P	1.11013500	-0.28441700	-1.23112100
C	-2.48287200	-0.28572900	0.49638200
C	-3.30565100	-1.08989200	-0.29772900
C	-2.78111900	1.08621100	0.61966400
C	-4.37267100	-0.53414700	-1.01380100
C	-3.83559100	1.64167400	-0.09005600
C	-4.63802500	0.83801000	-0.92151300
H	-4.97643700	-1.18335400	-1.63725300
H	-4.05958100	2.70148100	-0.01249000
H	-2.17732100	1.71887100	1.26322100
C	-1.86442900	-1.06345800	2.28386700
H	-0.90592800	-1.01293400	2.82547200
H	-2.48730200	-0.29513800	2.73400400
C	-2.48663000	-2.42357700	2.51558200
H	-3.36813400	-2.59466600	1.89593600
H	-3.11460700	-2.15100200	-0.40657200
C	-2.84041000	-2.63967600	3.98669800
C	-3.28828400	-4.07530500	4.33481200
H	-4.13405000	-4.02559600	5.03187700
H	-3.66591100	-4.57768300	3.43373400
O	-5.64071500	1.48160900	-1.57442300
C	-6.48910200	0.71768900	-2.43219100
H	-5.92062700	0.25533400	-3.24923400
H	-7.02595300	-0.06054500	-1.87497500
H	-7.20794500	1.42662200	-2.84761000
C	-2.17948500	-4.92465100	4.97229200
H	-1.83161200	-4.42143700	5.88550200
H	-1.31461100	-4.97023500	4.30061800
C	-2.64726800	-6.34058800	5.31629200
H	-2.99768500	-6.87017200	4.42268900
H	-1.83716100	-6.93195800	5.75984200
H	-3.47669800	-6.31725100	6.03471700
H	-1.98408200	-2.34774700	4.60816400

C	0.79291800	-1.34521300	-2.77419600
C	1.66427500	-3.10875000	-5.00111000
H	1.45586400	-3.78794600	-5.83875700
H	2.75591300	-3.00835500	-4.92664800
C	-1.06660900	-2.47727800	-4.08828100
H	-2.15716100	-2.57878800	-4.16327900
C	1.34879600	-0.77120600	-4.10072400
H	0.90423200	0.20722900	-4.30802500
H	2.43387000	-0.63473900	-4.03727300
C	1.09625800	-3.69251100	-3.69259800
H	1.55798600	-4.66711100	-3.48847000
C	-0.74496900	-1.53241300	-2.91217300
H	-1.14664500	-1.95683000	-1.98754700
H	-1.23712500	-0.56636200	-3.06807800
C	1.02408400	-1.73261500	-5.26756200
H	1.43272500	-1.30540200	-6.19225200
C	-0.43084700	-3.85444100	-3.81847400
H	-0.84034900	-4.28211700	-2.89452600
H	-0.66810100	-4.54698700	-4.63747100
C	1.43182900	-2.73950100	-2.52446200
H	2.51990300	-2.64521200	-2.44060700
H	1.06217300	-3.15814100	-1.58225200
C	-0.50355700	-1.88861900	-5.39565400
H	-0.74458700	-2.54694300	-6.24120200
H	-0.96585700	-0.91282800	-5.59969800
C	1.03888800	1.58663600	-1.52263500
C	-0.39603400	4.09833100	-0.83788400
H	-0.54029600	5.18491000	-0.90471500
H	-1.18452300	3.70347000	-0.18262000
C	1.97366100	3.70794600	-2.55226500
H	2.76360600	4.09425600	-3.20879100
C	-0.34623200	1.93274900	-2.12945900
H	-0.43941400	1.49019600	-3.12694700
H	-1.14728300	1.51555700	-1.50971600
C	0.99133700	3.78569400	-0.24333200
H	1.07524500	4.22623900	0.75850700
C	2.15404000	2.17706600	-2.42255500
H	3.13587300	1.97410100	-1.98246300
H	2.13542700	1.72159900	-3.41637800
C	-0.50992900	3.46325900	-2.23745200
H	-1.49838100	3.67857300	-2.66283900
C	2.08967600	4.35599300	-1.15992900
H	3.08084400	4.15441300	-0.73110500
H	1.98416700	5.44624000	-1.24034900
C	1.15722100	2.25624700	-0.12248100
H	0.38514200	1.86439500	0.55179400
H	2.13196400	2.01679700	0.32255600

C	0.59182100	4.02610900	-3.15602300
H	0.47299700	5.11225900	-3.26740400
H	0.50669900	3.58443800	-4.15844400
S	-1.64860100	-4.35190000	0.83401000
O	-1.47259400	-3.46213100	2.15468600
O	-0.77401400	-3.87722500	-0.23393200
O	-3.05807100	-4.62105500	0.56216600
C	-0.86063000	-5.91301700	1.50827500
F	0.24147600	-5.60882700	2.19250800
F	-0.54965500	-6.67927300	0.46643000
F	-1.72497800	-6.54501800	2.29552500
H	-3.64247500	-1.92311300	4.19957900
